# Supplementary material for: Meta-imputation of transcriptome from genotypes across multiple datasets by leveraging publicly available summary-level data
Source: PLoS Genet. 2022 Jan 31;18(1):e1009571. doi: 10.1371/journal.pgen.1009571 (PMC8830793; doi:10.1371/journal.pgen.1009571)
Supplement: S1 Text — (DOCX) [file pgen.1009571.s001.docx]

*Supporting Information for*

**Meta-imputation of transcriptome from genotypes across multiple datasets by leveraging publicly available summary-level data**

Andrew E. Liu^1^(*), Hyun Min Kang^1^(*)

^1^Department of Biostatistics and Center for Statistical Genetics, University of Michigan,
Ann Arbor, Michigan, United States of America

* [aeyliu@umich.edu](mailto:aeyliu@umich.edu) (AEL); * [hmkang@umich.edu](mailto:hmkang@umich.edu) (HMK)

# Supplementary Text

## Derivation of weights for SWAM

In this section we derive the equation for the weights in SWAM. We wish to impute expression for a reference sample of $N$ individuals with genotypes $\boldsymbol{X}_{\boldsymbol{t}}$ and measured tissue-specific expression $\boldsymbol{y}_{\boldsymbol{t}}$. Suppose we have single tissue imputation models for $K$ tissues, with $r\in\{1,\ldots,K\}$. For each gene $g$, we obtain a set of $K$ imputed expression levels ${\hat{\boldsymbol{s}}}_{\boldsymbol{j}}^{\boldsymbol{g}}=\boldsymbol{X}_{\boldsymbol{t}}\hat{\beta}_{j}^{g},$ with $j\in(1,\ldots,K)$. Dropping the superscript $g$ for convenience, we define$w=\left( w_{1},w_{2},\ldots,w_{K} \right)'$ be the set of weights corresponding to each of the tissues. The SWAM estimator is thus:

$${\hat{\boldsymbol{m}}}_{\boldsymbol{SWAM}}=\sum_{j=1}^{k} w_{j}{\hat{\boldsymbol{s}}}_{\boldsymbol{j}}$$

For further convenience, we denote ${\hat{\boldsymbol{m}}}_{SWAM}$ as $\hat{\boldsymbol{m}}.$ Then, for each gene separately, the values for $w$ are determined by minimizing the expression:

$$E\left[ \left\| \left. \hat{\boldsymbol{m}}-\boldsymbol{y}_{\boldsymbol{t}} \right\| \right._{2}^{2} \right]=E\left[ \left\| \left. \sum_{i=1}^{k} ({w_{i}\hat{\boldsymbol{s}}}_{i})-\boldsymbol{y}_{\boldsymbol{t}} \right\| \right._{2}^{2} \right]=E\left[ \sum_{i=1}^{k} w_{i}^{2}{({\hat{\boldsymbol{s}}}_{\boldsymbol{i}}-\boldsymbol{y}_{\boldsymbol{t}})}^{2} \right]$$

Without loss of generality, we set the constraint $\sum_{i=1}^{k} w_{i}=1$. The objective function to be minimized is

$$\mathcal{L}\left( \boldsymbol{w},\lambda\right)=E\left[ \left\| \left. \hat{\boldsymbol{m}}-\boldsymbol{y}_{\boldsymbol{t}} \right\| \right._{2}^{2} \right]+\gamma\left( \sum_{i=1}^{k} w_{i}-1 \right)=\sum_{i=1}^{k} w_{i}^{2}{E[({\hat{\boldsymbol{s}}}_{\boldsymbol{i}}-\boldsymbol{y}_{\boldsymbol{t}})}^{2}]+2\sum_{i=1}^{k} \sum_{j=1}^{i-1} w_{i}w_{j}E\left[ \left( {\hat{\boldsymbol{s}}}_{\boldsymbol{i}}-\boldsymbol{y}_{\boldsymbol{t}} \right)^{\boldsymbol{'}}({\hat{\boldsymbol{s}}}_{\boldsymbol{j}}-\boldsymbol{y}_{\boldsymbol{t}}\boldsymbol{)} \right]+\gamma\left( \sum_{i=1}^{k} w_{i}-1 \right)$$

The gradient of $\mathcal{L}\left( \boldsymbol{w},\lambda\right)$ is

$$\nabla\mathcal{L}\left( \boldsymbol{w},\lambda\right)=\left[ \begin{matrix} w_{i}{E[({\hat{\boldsymbol{s}}}_{\boldsymbol{i}}-\boldsymbol{y}_{\boldsymbol{t}})}^{2}]+2\sum_{j\neq i} w_{j}E\left[ ({\hat{\boldsymbol{s}}}_{\boldsymbol{i}}-\boldsymbol{y}_{\boldsymbol{t}}\boldsymbol{)'}({\hat{\boldsymbol{s}}}_{\boldsymbol{j}}-\boldsymbol{y}_{\boldsymbol{t}}\boldsymbol{)} \right]+\gamma\\ \sum_{j=1}^{k} w_{j}-1 \end{matrix} \right], i \in\{1,\cdots,K\}$$

Solving this system of equations, we obtain the optimal weighting minimizing the expected MSE across single tissue imputed expressions as

$$w_{i}=\frac{\left[ S^{-1}\boldsymbol{1} \right]_{i}}{\sum_{j=1}^{K} \left[ S^{-1}\boldsymbol{1} \right]_{j}}$$

Where $S=\left[ \begin{matrix} {E[({\hat{\boldsymbol{s}}}_{\boldsymbol{1}}-\boldsymbol{y}_{\boldsymbol{t}})}^{2}] & \cdots& E\left[ ({\hat{\boldsymbol{s}}}_{\boldsymbol{1}}-\boldsymbol{y}_{\boldsymbol{t}}\boldsymbol{)'}({\hat{\boldsymbol{s}}}_{\boldsymbol{K}}-\boldsymbol{y}_{\boldsymbol{t}}\boldsymbol{)} \right] \\ \vdots& \ddots& \vdots\\ E\left[ ({\hat{\boldsymbol{s}}}_{\boldsymbol{K}}-\boldsymbol{y}_{\boldsymbol{t}}\boldsymbol{)'}({\hat{\boldsymbol{s}}}_{\boldsymbol{1}}-\boldsymbol{y}_{\boldsymbol{t}}\boldsymbol{)} \right] & \cdots& {E[({\hat{\boldsymbol{s}}}_{\boldsymbol{K}}-\boldsymbol{y}_{\boldsymbol{t}})}^{2}] \end{matrix} \right]$ and $\boldsymbol{1}$ = (1,…,1)’

## Regularization of weights

The weights derived in the previous section provide an optimal solution to the expression $\underset{w^{g}}{\mathrm{argmin}} E\left[ \left\| \left. \hat{\boldsymbol{m}}-\boldsymbol{y}_{\boldsymbol{t}} \right\| \right._{2}^{2} \right]$. In the scenario in which the tissues are highly correlated with each other, the matrix ${cov\left( \hat{\boldsymbol{m}} \right)}^{-1}=\left[ \begin{matrix} \left\langle{\hat{\boldsymbol{s}}}_{\boldsymbol{1}},{\hat{\boldsymbol{s}}}_{\boldsymbol{1}} \right\rangle& \cdots& \left\langle{\hat{\boldsymbol{s}}}_{\boldsymbol{1}},{\hat{\boldsymbol{s}}}_{\boldsymbol{K}} \right\rangle\\ \vdots& \ddots& \vdots\\ \left\langle{\hat{\boldsymbol{s}}}_{\boldsymbol{K}},{\hat{\boldsymbol{s}}}_{\boldsymbol{1}} \right\rangle& \cdots& \left\langle{\hat{\boldsymbol{s}}}_{\boldsymbol{K}},{\hat{\boldsymbol{s}}}_{\boldsymbol{K}} \right\rangle\end{matrix} \right]^{-1}$is numerically unstable as the columns of $cov\left( \hat{\boldsymbol{m}} \right)$ are no longer linearly independent. This can lead to high weights assigned to irrelevant tissues and lower weights for relevant tissues. Furthermore, this may result in weights that are over-fitted to the noise of the data.

To correct for this, we added a diagonal matrix, $\lambda I$ prior to inverting the matrix $cov\left( \hat{\boldsymbol{m}} \right)$, giving us the solution $w^{g}=\left[ cov\left( \hat{\boldsymbol{m}} \right)+\lambda I \right]^{-1}\hat{cov}(\hat{\boldsymbol{m}},\boldsymbol{y}_{\boldsymbol{t}})$. To choose the correct value of $\lambda$, we tested the imputation accuracy of $\hat{\boldsymbol{m}}$ in our validation test set for a large range of $\lambda$. We found that imputation accuracy was low when $\lambda=0$, likely due to overfitted and the amplification of noise. Larger values of $\lambda$ yielded better results but ignored the correlation structure between tissues. We found empirically that $\lambda=3$ provided the best results in our real-data validation of SWAM, which we use as the default value in our software (S5 Fig). However, because this value may depend highly on the scale and normalization of the data, we also implemented an optional 10-fold cross-validation step in our software to determine the optimal $\lambda$ based on the input data. We also still provide users with an option that allows the user to manually specify the value of $\lambda$.

We also found that differences in within-tissue variability could lead to biased weights. As such, we applied a normalization step to ensure that each tissue’s predicted expression followed a standard normal distribution. As a result, our equation for optimal weights is equivalent to $w^{g}=\left[ cor\left( \hat{\boldsymbol{m}} \right)+\lambda I \right]^{-1}\hat{cor}(\hat{\boldsymbol{m}},\boldsymbol{y}_{\boldsymbol{t}})$, as seen in the Materials and Methods section.

## Application of SWAM to other target tissues

Throughout our work we primarily used the LCL tissue as our target tissue for application of SWAM. In addition to producing SWAM-LCL models, we also generated models targeting each of the 44 GTEx v6 tissues. S6 Fig displays the heatmap of weight contribution towards each of the tissues. The rows correspond to the SWAM model for each tissue type, and the color intensity of the columns show the contribution of each tissue towards the targeted tissue (number of times the tissue contributed the highest weight). Overall, we observe clustering that appears to separate the tissue types quite well. For example, brain tissues are primarily getting high weights from other brain tissues while receiving low weights from all other tissue types. This heatmap provides evidence of SWAM being able to capture tissue-specific signals.
