# Supplementary material for: Meta-imputation of transcriptome from genotypes across multiple datasets by leveraging publicly available summary-level data
Source: PLoS Genet. 2022 Jan 31;18(1):e1009571. doi: 10.1371/journal.pgen.1009571 (PMC8830793; doi:10.1371/journal.pgen.1009571)
Supplement: S1 Fig — The first panel shows how SWAM can be used to impute expression levels via prediXcan, while the second panel shows the required inputs to conduct TWAS via metaXcan. (PDF) [file pgen.1009571.s002.pdf]

Supplementary Figures

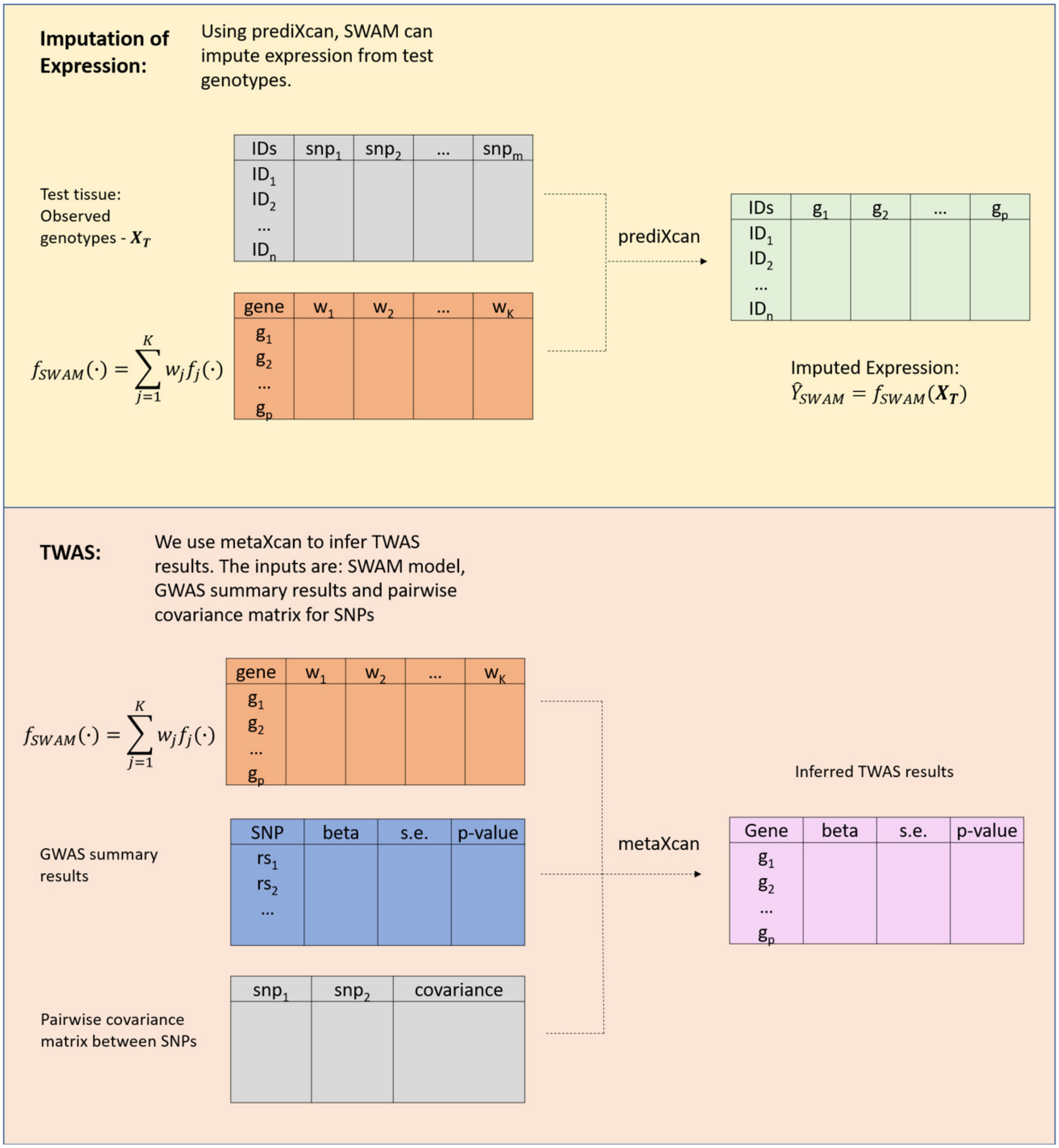

Supplementary Figure 1 – Using SWAM to impute expression and conduct TWAS

The first panel shows how SWAM can be used to impute expression levels via prediXcan, while the second panel shows the required inputs to conduct TWAS via metaXcan.
