# Supplementary material for: Meta-imputation of transcriptome from genotypes across multiple datasets by leveraging publicly available summary-level data
Source: PLoS Genet. 2022 Jan 31;18(1):e1009571. doi: 10.1371/journal.pgen.1009571 (PMC8830793; doi:10.1371/journal.pgen.1009571)
Supplement: S2 Fig — We expanded our simulation study to examine the effects of a larger number of causal variants, ranging from 5 to 125 variants, across a wide range of heritability levels. We found that increasing the number of causal variants had very little effect on the predictive performance of SWAM. We believe that this is because the expected imputation accuracy largely depends on the total heritability explained by the causal SNPs. (PDF) [file pgen.1009571.s003.pdf]

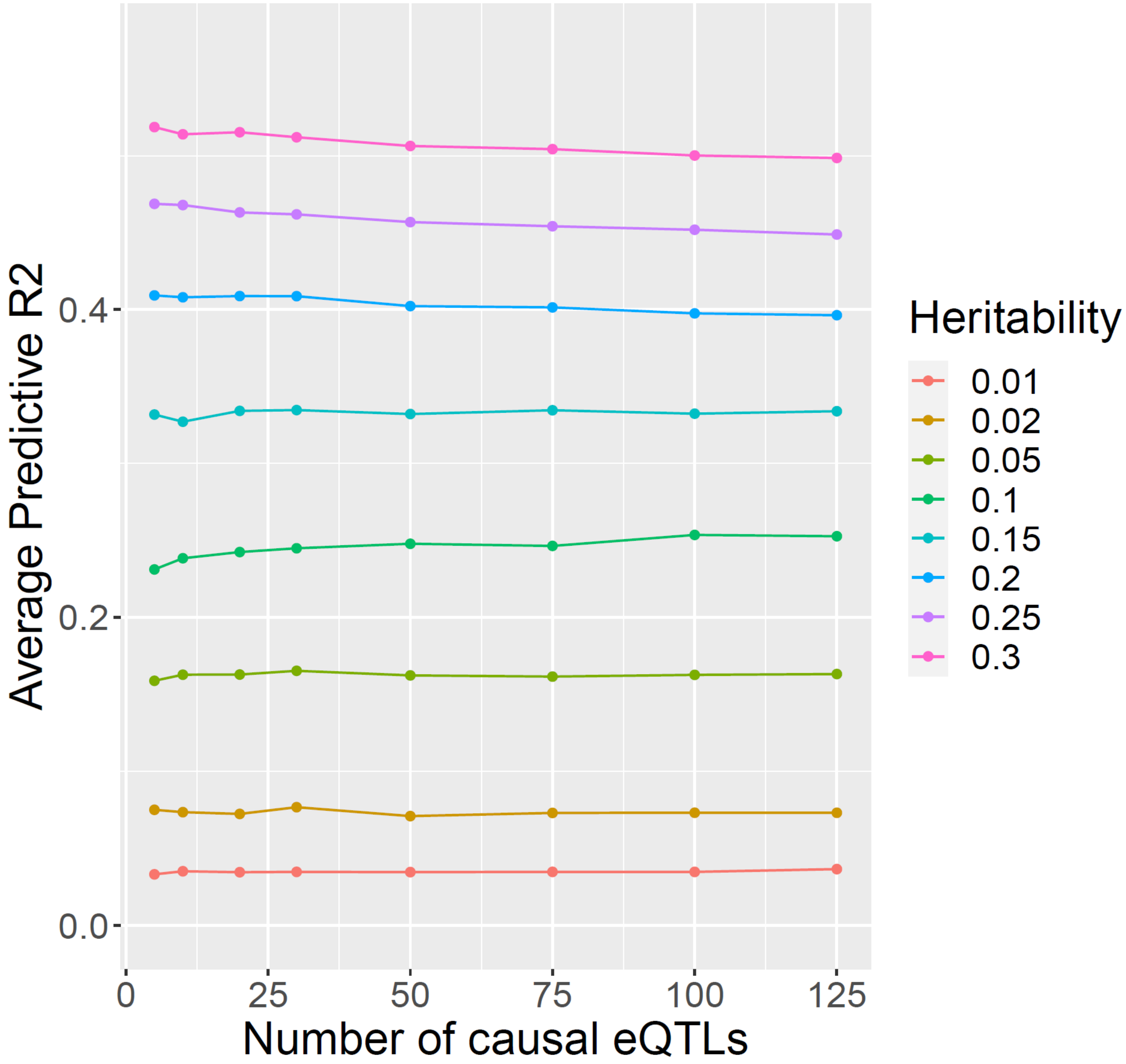

Supplementary Figure 2 – Impact of number of causal variants in simulation study

We expanded our simulation study to examine the effects of a larger number of causal variants, ranging from 5 to 125 variants, across a wide range of heritability levels. We found that increasing the number of causal variants had very little effect on the predictive performance of SWAM. We believe that this is because the expected imputation accuracy largely depends on the total heritability explained by the causal SNPs
