# Supplementary material for: Meta-imputation of transcriptome from genotypes across multiple datasets by leveraging publicly available summary-level data
Source: PLoS Genet. 2022 Jan 31;18(1):e1009571. doi: 10.1371/journal.pgen.1009571 (PMC8830793; doi:10.1371/journal.pgen.1009571)
Supplement: S3 Fig — The principal behind SWAM is it considers the bias-variance tradeoff for each tissue, and assigns higher weights to tissues that reduce MSE. In this example, tissues such as Skeletal Muscle have a high sample size (and therefore lower variance) but may be biased as they are not the relevant tissue to the tissue of interest (in this case LCL). Other tissues such as Fibroblasts may have a lower sample size but compensate by having low bias (high relevance to tissue of interest) and will contribute more weight. (PDF) [file pgen.1009571.s004.pdf]

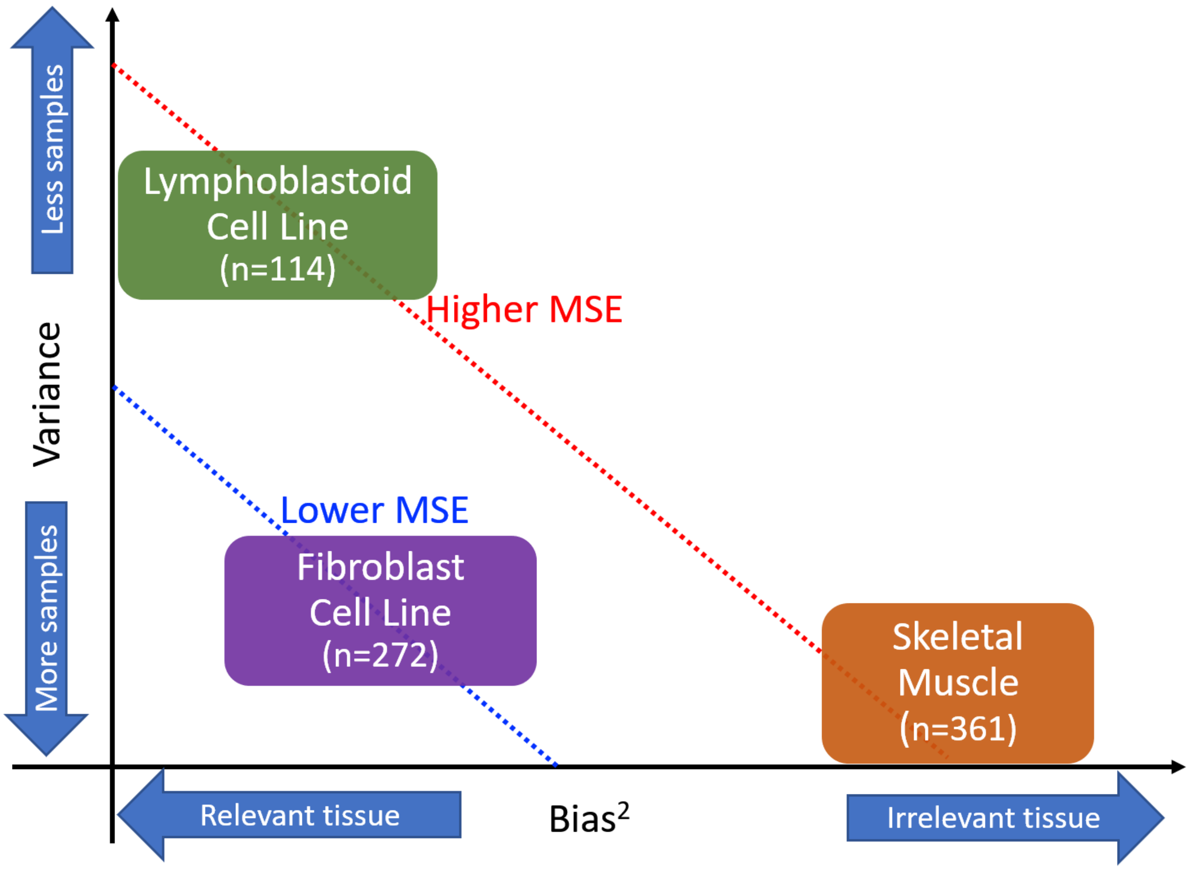

Supplementary Figure 3 – Bias-variance tradeoff for other tissues

The principal behind SWAM is it considers the bias-variance tradeoff for each tissue, and assigns higher weights to tissues that reduce MSE. In this example, tissues such as Skeletal Muscle have a high sample size (and therefore lower variance) but may be biased as they are not the relevant tissue to the tissue of interest (in this case LCL). Other tissues such as Fibroblasts may have a lower sample size but compensate by having low bias (high relevance to tissue of interest) and will contribute more weight.
