# Supplementary material for: Meta-imputation of transcriptome from genotypes across multiple datasets by leveraging publicly available summary-level data
Source: PLoS Genet. 2022 Jan 31;18(1):e1009571. doi: 10.1371/journal.pgen.1009571 (PMC8830793; doi:10.1371/journal.pgen.1009571)
Supplement: S4 Fig — (A) shows the ERAP2 gene, which had a single tissue r2 = 0.854, while the SWAM model had r2 = 0.812. (B) depicts the scenario where SWAM is able to leverage information from other tissues to make up for the relatively lower quality of the target tissue. here the single tissue model gave r2 = 0.125 while SWAM increased the accuracy to r2 = 0.492. (C) shows an example where the eQTLs are highly tissue specific. Here, SWAM improved the single tissue accuracy from r2 = 0.077 to r2 = 0.323. (PDF) [file pgen.1009571.s005.pdf]

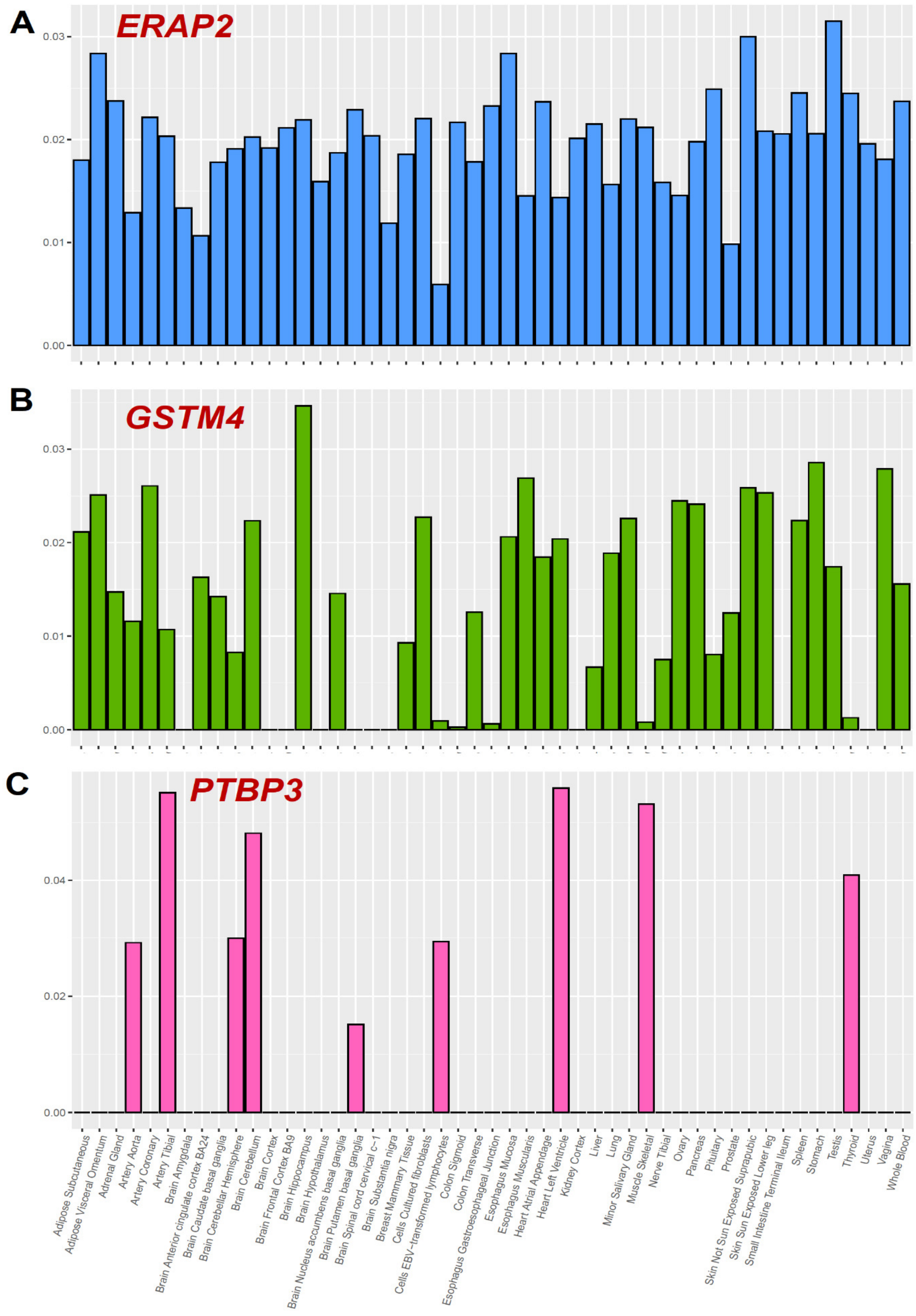

Supplementary Figure 4 – The distribution of weights for SWAM for three selected genes.

(A) shows the ERAP2 gene, which had a single tissue r^2 = 0.854, while the SWAM model had r^2 = 0.812. (B) depicts the scenario where SWAM is able to leverage information from other tissues to make up for the relatively lower quality of the target tissue – here the single tissue model gave r^2 = 0.125 while SWAM increased the accuracy to r^2 = 0.492. (C) shows an example where the eQTLs are highly tissue specific. Here, SWAM improved the single tissue accuracy from r^2 = 0.077 to r^2 = 0.323.
