# Supplementary material for: Meta-imputation of transcriptome from genotypes across multiple datasets by leveraging publicly available summary-level data
Source: PLoS Genet. 2022 Jan 31;18(1):e1009571. doi: 10.1371/journal.pgen.1009571 (PMC8830793; doi:10.1371/journal.pgen.1009571)
Supplement: S5 Fig — We calibrated our tuning parameter using GEUVADIS data as our external validation. As a result, we set the default value of this tuning parameter to 3 in our software, which we believe performs best. However, as the parameter may depend on the scaling and normalization of the data, there is also an option to calculate the tuning parameter via cross-validation. (PDF) [file pgen.1009571.s006.pdf]

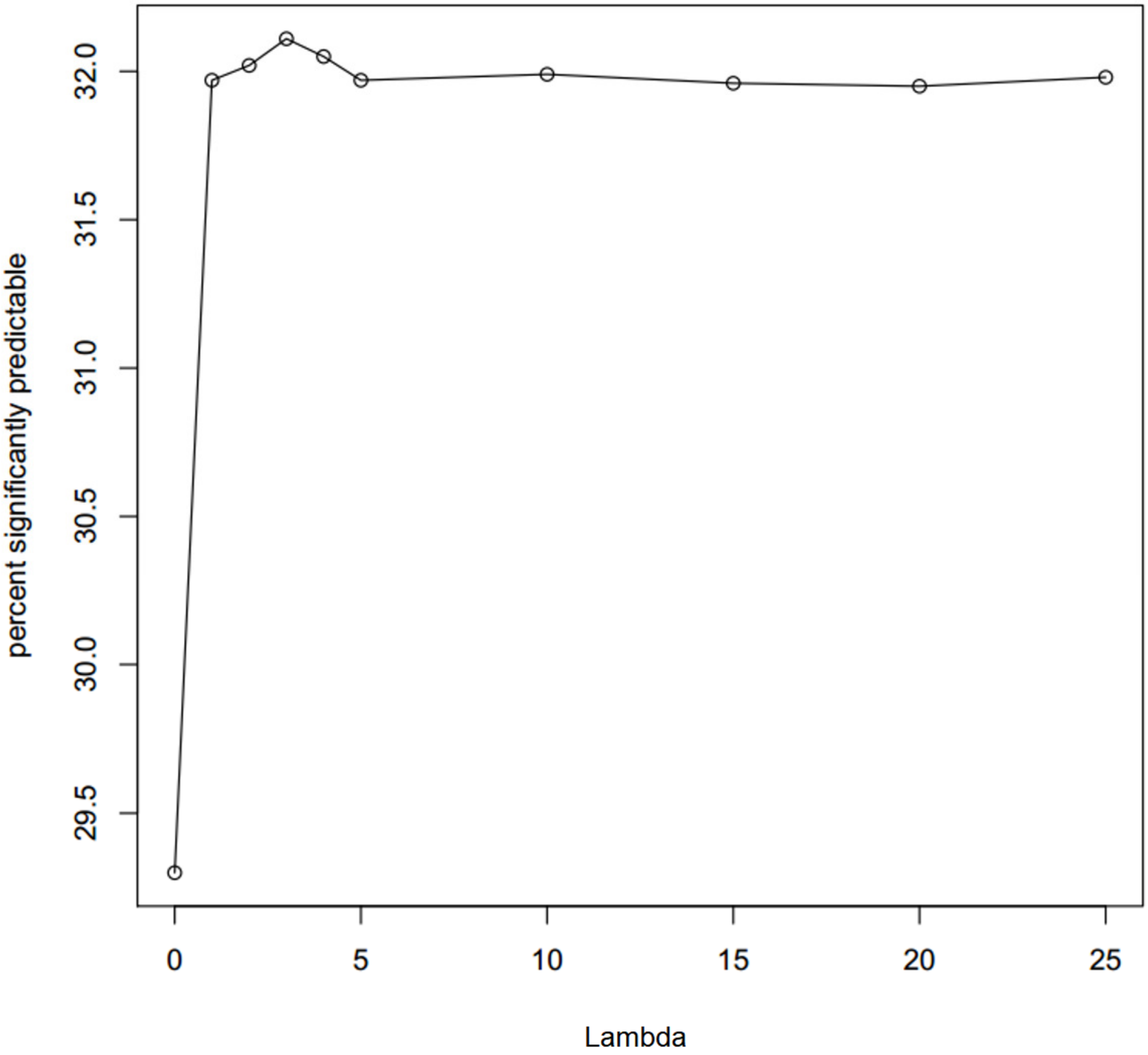

Supplementary Figure 5 – Calibration of tuning parameter for SWAM using empirical data

We calibrated our tuning parameter using GEUVADIS data as our external validation. As a result, we set the default value of this tuning parameter to 3 in our software, which we believe performs best. However, as the parameter may depend on the scaling and normalization of the data, there is also an option to calculate the tuning parameter via cross-validation.
