# Supplementary material for: Meta-imputation of transcriptome from genotypes across multiple datasets by leveraging publicly available summary-level data
Source: PLoS Genet. 2022 Jan 31;18(1):e1009571. doi: 10.1371/journal.pgen.1009571 (PMC8830793; doi:10.1371/journal.pgen.1009571)
Supplement: S6 Fig — Here, we used SWAM to derive multi-tissue imputation models for all 44 GTEx v6 tissues. Each cell in this heatmap depicts the number of times each tissue contributed the highest weight to the target tissue. Here, the rows correspond to the target tissue and the columns correspond to the weight contribution of each tissue. For the sake of clarity, the diagonal values were not included as they were consistently much higher than the remaining elements of the matrix. (PDF) [file pgen.1009571.s007.pdf]

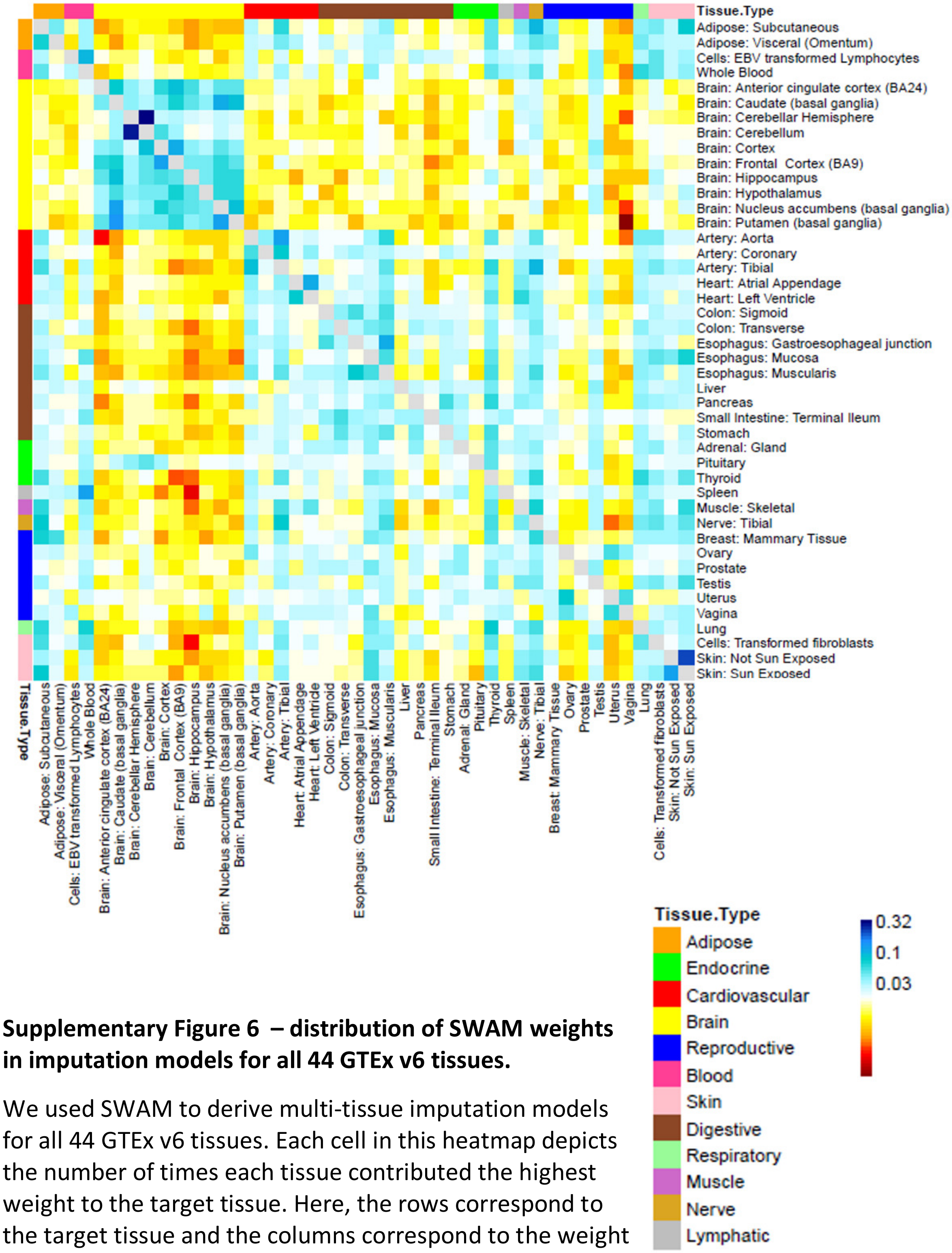

Supplementary Figure 6 – distribution of SWAM weights in imputation models for all 44 GTEx v6 tissues.

We used SWAM to derive multi-tissue imputation models for all 44 GTEx v6 tissues. Each cell in this heatmap depicts the number of times each tissue contributed the highest weight to the target tissue. Here, the rows correspond to the target tissue and the columns correspond to the weight contribution of each tissue. For the sake of clarity, the diagonal values were not included as they were consistently much higher than the remaining elements of the matrix.
