# Supplementary material for: Meta-imputation of transcriptome from genotypes across multiple datasets by leveraging publicly available summary-level data
Source: PLoS Genet. 2022 Jan 31;18(1):e1009571. doi: 10.1371/journal.pgen.1009571 (PMC8830793; doi:10.1371/journal.pgen.1009571)
Supplement: S7 Fig — We performed a comprehensive simulation study identical to the one displayed in Fig 2, but with only 2 causal variants instead of 10. We find that the adjusting the number of causal variants does not affect the results in any substantial way, which we believe is due to the fact that heritability was fixed regardless of the number of variants used. (PDF) [file pgen.1009571.s008.pdf]

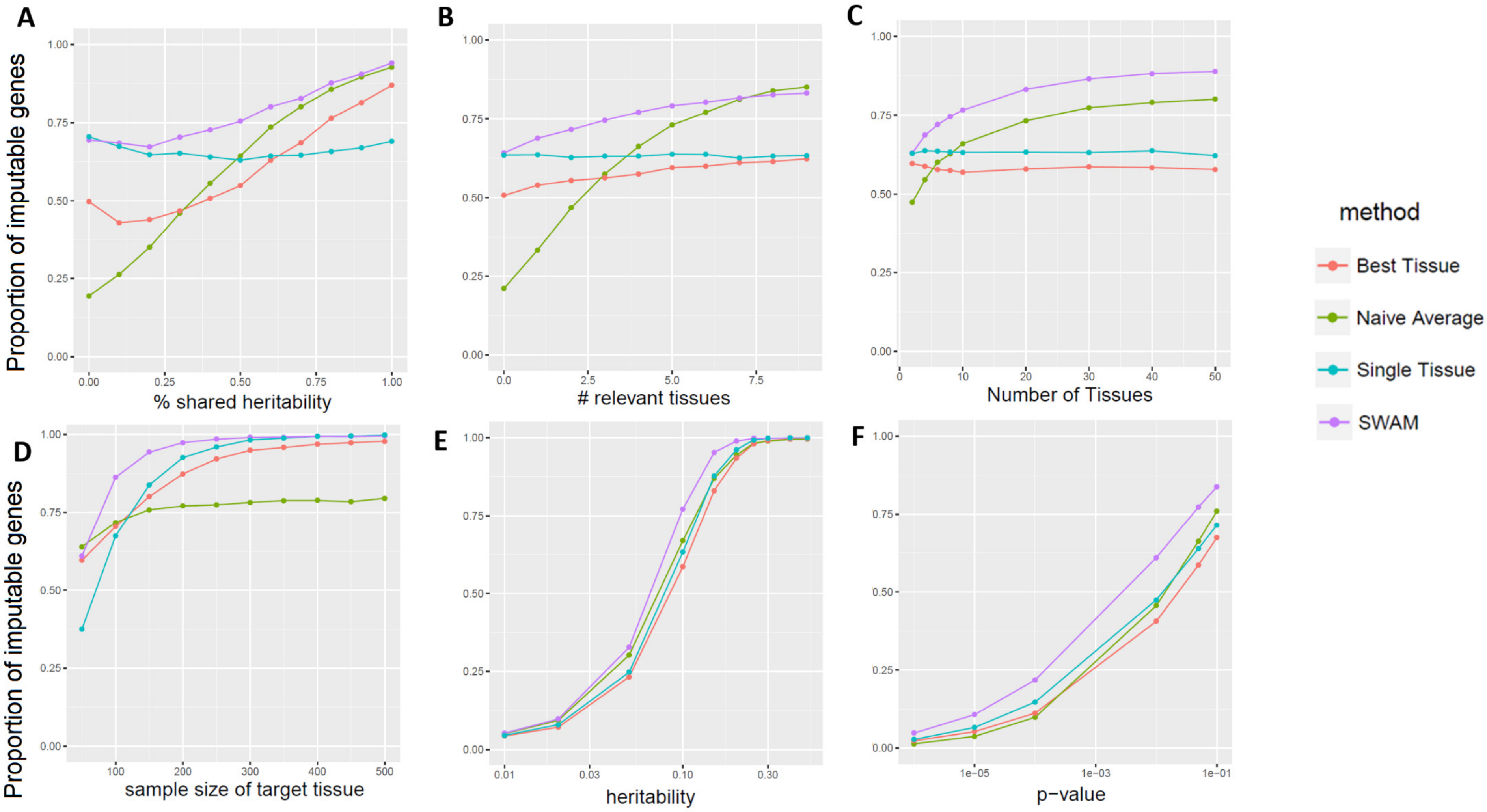

Supplementary Figure 7 – Simulation study with only 2 causal variants

We performed a comprehensive simulation study identical to the one displayed in Figure 2, but with only 2 causal variants instead of 10. We find that the adjusting the number of causal variants does not affect the results in any substantial way, which we believe is due to the fact that heritability was fixed regardless of the number of variants used.
