# Supplementary material for: Meta-imputation of transcriptome from genotypes across multiple datasets by leveraging publicly available summary-level data
Source: PLoS Genet. 2022 Jan 31;18(1):e1009571. doi: 10.1371/journal.pgen.1009571 (PMC8830793; doi:10.1371/journal.pgen.1009571)
Supplement: S8 Fig — Here we display the Q-Q plot of p-values before and after controlling for genomic inflation in our Liver-LDL and liver-T2D TWAS. The LDL plot suggests an enrichment in signals due to high power in the original GWAS analysis. (PDF) [file pgen.1009571.s009.pdf]

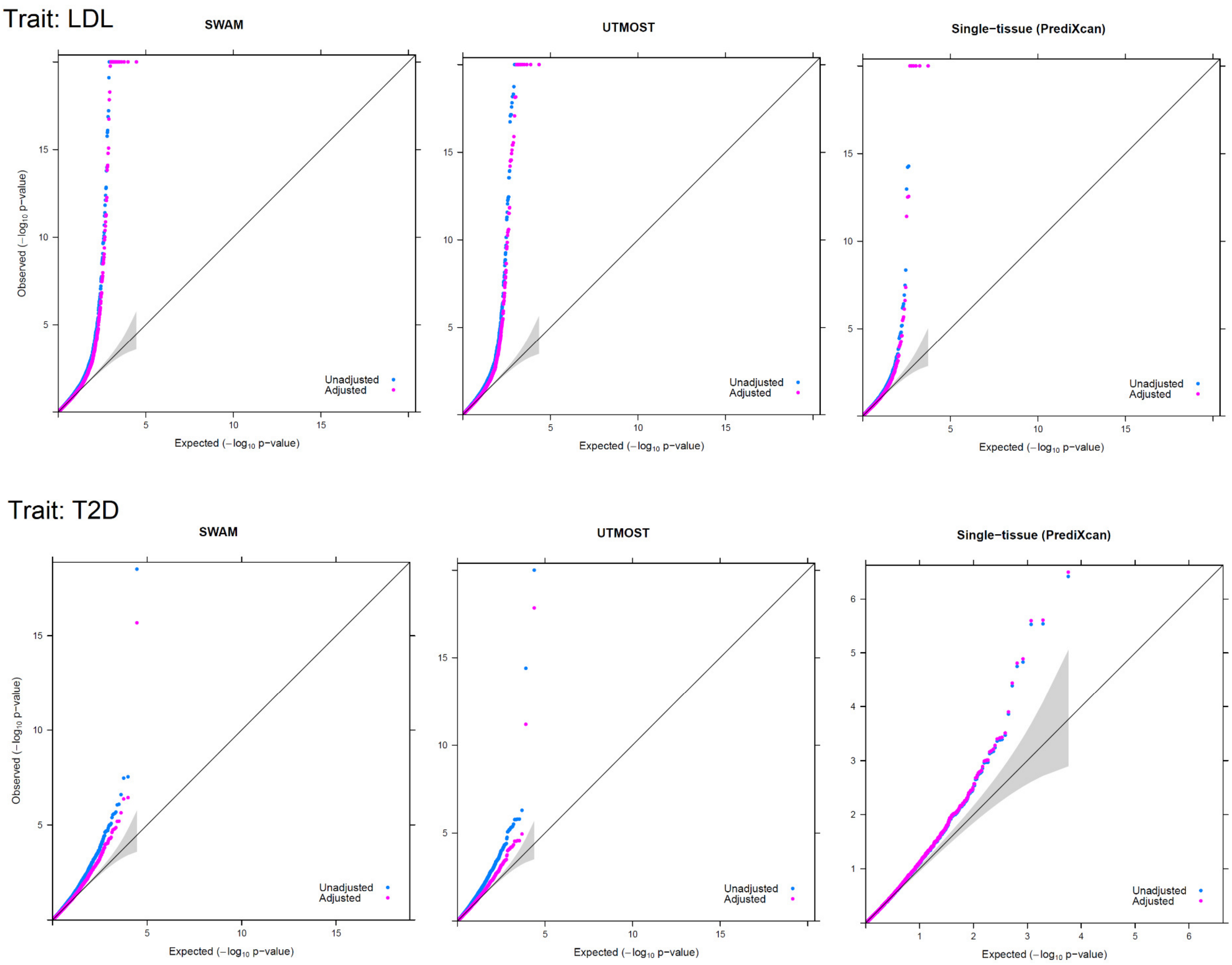

Supplementary Figure 8 – Q-Q plot of p-values for Liver tissue in LDL and T2D TWAS

Here we display the Q-Q plot of p-values before and after controlling for genomic inflation in our Liver-LDL and liver-T2D TWAS. The LDL plot suggests an enrichment in signals due to high power in the original GWAS analysis.
