# Supplementary material for: Meta-imputation of transcriptome from genotypes across multiple datasets by leveraging publicly available summary-level data
Source: PLoS Genet. 2022 Jan 31;18(1):e1009571. doi: 10.1371/journal.pgen.1009571 (PMC8830793; doi:10.1371/journal.pgen.1009571)
Supplement: S1 Table — We tested type 1 error rates by training different methods on simulated data and comparing predicted expression concordance with simulated null test data (gene expression generated with zero genetic effect). Here, we show the proportion of significantly imputable genes (false positives rate) by p-value threshold. (PDF) [file pgen.1009571.s010.pdf]

P-value Threshold	Proportion of Significantly Imputable Genes by Method (False Positives)				
	Single Tissue	Naïve Average	Best Tissue	SWAM	UTMOST
0.1	0.1007	0.0994	0.1021	0.0959	0.0972
0.05	0.0511	0.0509	0.0488	0.0492	0.048
0.01	0.0092	0.0104	0.011	0.0093	0.0102
0.001	0.0006	0.0012	0.0009	0.0007	0.0012
0.0001	<0.0001	<0.0001	<0.0001	<0.0001	0.0001
0.00001	<0.0001	<0.0001	<0.0001	<0.0001	<0.0001
0.000001	<0.0001	<0.0001	<0.0001	<0.0001	<0.0001

Supplementary Table 1 – Evaluation of Type I errors across different methods using simulated expression and genotype data.

We tested type 1 error rates by training different methods on simulated data and comparing predicted expression concordance with simulated null test data (gene expression generated with zero genetic effect). Here, we show the proportion of significantly imputable genes (false positives rate) by p-value threshold.
