# Supplementary material for: Meta-imputation of transcriptome from genotypes across multiple datasets by leveraging publicly available summary-level data
Source: PLoS Genet. 2022 Jan 31;18(1):e1009571. doi: 10.1371/journal.pgen.1009571 (PMC8830793; doi:10.1371/journal.pgen.1009571)
Supplement: S2 Table — Counts (B-H counts) are based on Benjamini-Hochberg procedure false discovery rate of 0.05. The last column displays the number of counts at p-value threshold 0.05 (without any corrections). (PDF) [file pgen.1009571.s011.pdf]

Method	Sample Size	Total # genes	Genes with FDR < 0.05	P-value threshold for FDR=0.05	Genes with p-value < 0.05
DGN Whole Blood	922	10488	2390	0.009018	3450
Adipose Subcutaneous	298	6410	1500	0.005667	2203
Adipose Visceral Omentum	185	4044	963	0.003619	1432
Adrenal Gland	126	3592	735	0.002755	1151
Artery Aorta	197	5497	1177	0.004426	1783
Artery Coronary	118	2890	616	0.00231	926
Artery Tibial	285	6469	1439	0.005399	2124
Brain Anterior cingulate cortex BA24	72	2320	322	0.00117	580
Brain Caudate basal ganglia	100	3171	528	0.001979	895
Brain Cerebellar Hemisphere	89	3730	575	0.002172	1002
Brain Cerebellum	103	4211	661	0.002498	1144
Brain Cortex	96	3129	511	0.00193	878
Brain Frontal Cortex BA9	92	2785	456	0.001699	770
Brain Hippocampus	81	2228	336	0.001254	572
Brain Hypothalamus	81	2152	339	0.001264	554
Brain Nucleus accumbens basal ganglia	93	2711	454	0.001709	755
Brain Putamen basal ganglia	82	2465	391	0.001461	655
Breast Mammary Tissue	183	4119	933	0.003455	1430
Cells EBV-transformed lymphocytes	114	3661	1552	0.005845	1943
Cells Transformed fibroblasts	272	6966	1690	0.00635	2451
Colon Sigmoid	124	3408	680	0.002391	1052
Colon Transverse	169	4300	1013	0.003803	1536
Esophagus Gastroesophageal Junction	127	3325	716	0.002689	1101
Esophagus Mucosa	241	6136	1469	0.00553	2148
Esophagus Muscularis	218	5774	1272	0.004808	1959
Heart Atrial Appendage	159	3994	849	0.003167	1323
Heart Left Ventricle	190	4360	942	0.003537	1451
Liver	97	2583	427	0.001547	719
Lung	278	5736	1355	0.005122	1985
Muscle Skeletal	361	5963	1197	0.004454	1876
Nerve Tibial	256	7114	1450	0.005483	2186
Ovary	85	2430	417	0.001566	689
Pancreas	149	4226	911	0.003426	1406
Pituitary	87	2903	496	0.001875	808
Prostate	87	2275	398	0.001421	629
Skin Not Sun Exposed Suprapubic	196	5050	1063	0.003997	1619
Skin Sun Exposed Lower leg	302	6656	1463	0.005489	2145
Small Intestine Terminal Ileum	77	2407	450	0.001694	735
Spleen	89	3312	759	0.002821	1166

Stomach	170	3750	878	0.003291	1350
Testis	157	5893	956	0.003607	1563
Thyroid	278	7126	1454	0.005469	2208
Uterus	70	1919	297	0.001071	521
Vagina	79	1813	307	0.001149	511
Whole Blood	338	6178	1427	0.005343	2106

Supplementary Table 2 – GTEx version 6 comparisons of single-tissue and multi-tissue imputation models using GEUVADIS LCL RNA-Seq expression as validation.

Counts (B-H counts) are based on Benjamini-Hochberg procedure false discovery rate of 0.05. The last column displays the number of counts at p-value threshold 0.05 (without any corrections)
