# Supplementary material for: Meta-imputation of transcriptome from genotypes across multiple datasets by leveraging publicly available summary-level data
Source: PLoS Genet. 2022 Jan 31;18(1):e1009571. doi: 10.1371/journal.pgen.1009571 (PMC8830793; doi:10.1371/journal.pgen.1009571)
Supplement: S3 Table — Counts (B-H counts) are based on Benjamini-Hochberg procedure false discovery rate of 0.05. The last column displays the number of counts at p-value threshold 0.05 (without any corrections). (PDF) [file pgen.1009571.s012.pdf]

Method	Sample Size	Total # genes	Genes with FDR < 0.05	P-value threshold for FDR=0.05	Genes with p-value < 0.05
Adipose Subcutaneous	328	6689	2259	0.016828	2739
Adipose Visceral Omentum	273	5286	1905	0.017961	2276
Adrenal Gland	146	3784	1322	0.017403	1556
Artery Aorta	236	5488	1850	0.01685	2217
Artery Coronary	128	2838	1040	0.018238	1193
Artery Tibial	329	6836	2131	0.015564	2642
Brain Amygdala	81	1876	581	0.015227	690
Brain Anterior cingulate cortex BA24	102	2628	838	0.01577	1016
Brain Caudate basal ganglia	126	3272	1035	0.015801	1261
Brain Cerebellar Hemisphere	113	3810	1050	0.013652	1310
Brain Cerebellum	137	4899	1310	0.013357	1667
Brain Cortex	119	3422	1073	0.015676	1317
Brain Frontal Cortex BA9	104	2812	879	0.015504	1072
Brain Hippocampus	99	2217	708	0.015749	836
Brain Hypothalamus	98	2219	710	0.015917	862
Brain Nucleus accumbens basal ganglia	114	2820	921	0.016269	1117
Brain Putamen basal ganglia	98	2542	806	0.015838	978
Brain Spinal cord cervical c-1	76	2003	600	0.014347	744
Brain Substantia nigra	70	1609	496	0.015396	586
Breast Mammary Tissue	211	4280	1622	0.018909	1900
Cells EBV-transformed lymphocytes	96	2777	1731	0.030999	1808
Cells Transformed fibroblasts	256	6297	2336	0.018541	2748
Colon Sigmoid	185	4257	1556	0.018221	1850
Colon Transverse	210	4457	1771	0.019685	2040
Esophagus Gastroesophageal Junction	185	4325	1603	0.018478	1852
Esophagus Mucosa	307	6744	2305	0.017054	2789
Esophagus Muscularis	287	6354	2119	0.016546	2574
Heart Atrial Appendage	231	4866	1675	0.017206	2015
Heart Left Ventricle	233	4449	1491	0.016605	1809
Liver	134	2746	883	0.015909	1062
Lung	333	6251	2190	0.017508	2636
Minor Salivary Gland	74	1785	652	0.018105	749
Muscle Skeletal	421	6323	1901	0.015017	2362
Nerve Tibial	305	7512	2179	0.014492	2767
Ovary	99	2406	818	0.016888	954
Pancreas	180	4369	1504	0.01713	1786
Pituitary	143	3691	1228	0.016612	1485
Prostate	114	2487	926	0.018601	1069
Skin Not Sun Exposed Suprapubic	285	6092	1997	0.016387	2423

Skin Sun Exposed Lower leg	359	7221	2201	0.015187	2789
Small Intestine Terminal Ileum	103	2482	991	0.019855	1121
Spleen	119	3753	1514	0.020099	1726
Stomach	200	3899	1518	0.019332	1748
Testis	191	5919	1451	0.012238	1889
Thyroid	344	7556	2249	0.014839	2812
Uterus	82	1957	681	0.017	800
Vagina	91	1889	690	0.01778	790
Whole Blood	315	5432	1915	0.01758	2288

Supplementary Table 3 – GTEx version 7 comparisons of single-tissue and multi-tissue imputation models using GEUVADIS LCL RNA-Seq expression as validation.

Counts (B-H counts) are based on Benjamini-Hochberg procedure false discovery rate of 0.05. The last column displays the number of counts at p-value threshold 0.05 (without any corrections)
