# Supplementary material for: Meta-imputation of transcriptome from genotypes across multiple datasets by leveraging publicly available summary-level data
Source: PLoS Genet. 2022 Jan 31;18(1):e1009571. doi: 10.1371/journal.pgen.1009571 (PMC8830793; doi:10.1371/journal.pgen.1009571)
Supplement: S4 Table — Counts (B-H counts) are based on Benjamini-Hochberg procedure false discovery rate of 0.05. The last column displays the number of counts at p-value threshold 0.05 (without any corrections). (PDF) [file pgen.1009571.s013.pdf]

Method	Sample Size	Total # genes	Genes with FDR < 0.05	P-value threshold for FDR=0.05	Genes with p-value < 0.05
Adipose Subcutaneous	581	6847	2114	0.015316	2662
Adipose Visceral Omentum	469	5769	1957	0.016922	2381
Adrenal Gland	233	3816	1279	0.016704	1520
Artery Aorta	387	6090	1844	0.015047	2286
Artery Coronary	213	3177	1119	0.01758	1330
Artery Tibial	584	6955	2023	0.014432	2545
Brain Amygdala	129	2081	682	0.016269	806
Brain Anterior cingulate cortex BA24	147	2662	840	0.015751	1030
Brain Caudate basal ganglia	194	3795	1146	0.015054	1430
Brain Cerebellar Hemisphere	175	4482	1127	0.012532	1499
Brain Cerebellum	209	5254	1299	0.012294	1727
Brain Cortex	205	4169	1203	0.014418	1520
Brain Frontal Cortex BA9	175	3424	1019	0.014767	1299
Brain Hippocampus	165	2806	886	0.015723	1071
Brain Hypothalamus	170	2742	911	0.016563	1076
Brain Nucleus accumbens basal ganglia	202	3629	1076	0.014803	1357
Brain Putamen basal ganglia	170	3365	1035	0.015336	1266
Brain Spinal cord cervical c-1	126	2455	744	0.015097	909
Brain Substantia nigra	114	1892	570	0.015031	695
Breast Mammary Tissue	396	5076	1756	0.017283	2114
Cells EBV-transformed lymphocytes	147	2537	1620	0.031497	1690
Cells Transformed fibroblasts	483	7421	2428	0.016349	2997
Colon Sigmoid	318	4847	1633	0.016741	1931
Colon Transverse	368	4923	1781	0.017988	2123
Esophagus Gastroesophageal Junction	330	4964	1675	0.016674	2029
Esophagus Mucosa	497	6872	2167	0.015721	2694
Esophagus Muscularis	465	6554	2030	0.015468	2530
Heart Atrial Appendage	372	5262	1696	0.016097	2085
Heart Left Ventricle	386	4902	1569	0.015841	1879
Kidney Cortex	73	1205	344	0.013871	411
Liver	208	2983	976	0.016303	1154
Lung	515	6173	2071	0.016651	2543
Minor Salivary Gland	144	2161	842	0.019464	944
Muscle Skeletal	706	6261	1762	0.014048	2251
Nerve Tibial	532	7764	2051	0.013198	2666
Ovary	167	2751	909	0.016349	1083
Pancreas	305	4710	1532	0.016218	1886
Pituitary	237	4262	1381	0.016124	1712
Prostate	221	3205	1125	0.017494	1333

Skin Not Sun Exposed Suprapubic	517	6802	2011	0.014719	2546
Skin Sun Exposed Lower leg	605	7203	2071	0.014317	2677
Small Intestine Terminal Ileum	174	2844	1145	0.019928	1302
Spleen	227	4527	1720	0.018922	2035
Stomach	324	4064	1542	0.018783	1802
Testis	322	6470	1518	0.01172	2070
Thyroid	574	7468	2130	0.01421	2737
Uterus	129	1944	699	0.017885	818
Vagina	141	1919	725	0.01884	832
Whole Blood	670	6195	2082	0.016804	2531

Supplementary Table 4 – GTEx version 8 comparisons of single-tissue and multi-tissue imputation models using GEUVADIS LCL RNA-Seq expression as validation.

Counts (B-H counts) are based on Benjamini-Hochberg procedure false discovery rate of 0.05. The last column displays the number of counts at p-value threshold 0.05 (without any corrections)
