# Supplementary material for: Meta-imputation of transcriptome from genotypes across multiple datasets by leveraging publicly available summary-level data
Source: PLoS Genet. 2022 Jan 31;18(1):e1009571. doi: 10.1371/journal.pgen.1009571 (PMC8830793; doi:10.1371/journal.pgen.1009571)
Supplement: S5 Table — We applied SWAM to all combinations of GTEx and DGN resources. For the GTEx resources, we always used every tissue available. In version 6, this comprised of 44 tissues. For version 7, there were 48 tissues and version 8 contained 49 tissues. For the sake of consistency, our target tissue for each of these combinations was GTEx v6 LCL. (PDF) [file pgen.1009571.s014.pdf]

Method	Total # genes	Genes with FDR < 0.05	P-value threshold for FDR=0.05	Genes with p-value < 0.05
SWAM-LCL (GTEx v6)	13213	3040	0.01145	4148
SWAM-LCL (GTEx v6 + DGN)	13213	3192	0.012077	4301
SWAM-LCL (GTEx v7)	13213	3060	0.011463	4215
SWAM-LCL (GTEx v7 + DGN)	13213	3411	0.012903	4469
SWAM-LCL (GTEx v8)	13213	3203	0.01212	4236
SWAM-LCL (GTEx v8 + DGN)	13213	3449	0.013046	4460
SWAM-LCL (GTEx v6 + v7)	13213	3283	0.012383	4385
SWAM-LCL (GTEx v6 + v7 + DGN)	13213	3361	0.012674	4480
SWAM-LCL (GTEx v6 + v8)	13213	3134	0.01185	4274
SWAM-LCL (GTEx v6 + v8 + DGN)	13213	3275	0.012389	4384
SWAM-LCL (GTEx v7 + v8)	13213	3259	0.01227	4326
SWAM-LCL (GTEx v7 + v8 + DGN)	13213	3368	0.012737	4478
SWAM-LCL (GTEx v6 + v7 + v8)	13213	3342	0.012641	4448
SWAM-LCL (GTEx v6 + v7 + v8 + DGN)	13213	3413	0.012878	4526
UTMOST-LCL	13213	2238	0.008466	3185
NAIVE AVERAGE	13213	2666	0.010066	3830
BEST TISSUE	13213	2493	0.009394	3663

Supplementary Table 5 – Comparison of all multi-tissue methods

We applied SWAM to all combinations of GTEx and DGN resources. For the GTEx resources, we always used every tissue available. In version 6, this comprised of 44 tissues. For version 7, there were 48 tissues and version 8 contained 49 tissues. For the sake of consistency, our target tissue for each of these combinations was GTEx v6 LCL.
