# Supplementary material for: Meta-imputation of transcriptome from genotypes across multiple datasets by leveraging publicly available summary-level data
Source: PLoS Genet. 2022 Jan 31;18(1):e1009571. doi: 10.1371/journal.pgen.1009571 (PMC8830793; doi:10.1371/journal.pgen.1009571)
Supplement: S6 Table — We used SWAM to derive an tissue-specific model for every GTEx version 6 tissue, and used these models as inputs to metaXcan to infer TWAS results. As mentioned in the Materials and Methods section, the HDL and LDL traits were from Global Lipids Genetics Consortium (GLGC) and Type-2 Diabetes (T2D) from the DIAGRAM consortium. (PDF) [file pgen.1009571.s015.pdf]

Tissue	HDL			LDL			T2D		
	# sig genes	p-value threshold	total genes	# sig genes	p-value threshold	total genes	# sig genes	p-value threshold	total genes
Adipose Subcutaneous	68 (24)	3.23E-06	15501	72 (10)	3.22E-06	15507	5 (2)	3.19E-06	15669
Adipose Visceral Omentum	69 (14)	3.26E-06	15326	63 (7)	3.26E-06	15333	6 (2)	3.23E-06	15476
Adrenal Gland	56 (9)	3.34E-06	14961	69 (12)	3.34E-06	14973	8 (2)	3.31E-06	15110
Artery Aorta	60 (17)	3.34E-06	14990	70 (20)	3.33E-06	15000	5 (3)	3.31E-06	15125
Artery Coronary	68 (6)	3.33E-06	15001	59 (6)	3.33E-06	15009	2 (1)	3.30E-06	15147
Artery Tibial	65 (15)	3.33E-06	14994	71 (16)	3.33E-06	15001	4 (2)	3.30E-06	15145
Brain Anterior cingulate cortex BA24	72 (3)	3.36E-06	14892	65 (7)	3.36E-06	14901	4 (0)	3.32E-06	15061
Brain Caudate basal ganglia	72 (8)	3.29E-06	15216	71 (12)	3.28E-06	15221	5 (1)	3.25E-06	15372
Brain Cerebellar Hemisphere	69 (8)	3.39E-06	14742	64 (10)	3.39E-06	14755	3 (2)	3.36E-06	14891
Brain Cerebellum	57 (13)	3.34E-06	14991	73 (17)	3.33E-06	15006	3 (2)	3.30E-06	15150
Brain Cortex	68 (11)	3.29E-06	15198	88 (15)	3.29E-06	15208	2 (1)	3.25E-06	15366
Brain Frontal Cortex BA9	63 (5)	3.32E-06	15061	61 (10)	3.32E-06	15073	4 (1)	3.28E-06	15239
Brain Hippocampus	73 (5)	3.31E-06	15098	66 (8)	3.31E-06	15106	3 (1)	3.27E-06	15269
Brain Hypothalamus	56 (3)	3.28E-06	15265	69 (6)	3.27E-06	15274	5 (1)	3.24E-06	15419
Brain Nucleus accumbens basal ganglia	65 (5)	3.30E-06	15152	77 (12)	3.30E-06	15166	4 (1)	3.27E-06	15313
Brain Putamen basal ganglia	75 (4)	3.35E-06	14926	74 (10)	3.35E-06	14935	6 (2)	3.32E-06	15066
Breast Mammary Tissue	69 (15)	3.19E-06	15682	70 (9)	3.19E-06	15687	6 (2)	3.16E-06	15838
Cells EBV-transformed lymphocytes	65 (12)	3.75E-06	13344	61 (9)	3.75E-06	13347	3 (1)	3.70E-06	13504
Cells Transformed fibroblasts	54 (23)	3.55E-06	14091	64 (24)	3.55E-06	14098	5 (4)	3.51E-06	14253
Colon Sigmoid	60 (4)	3.31E-06	15124	70 (9)	3.30E-06	15138	6 (1)	3.27E-06	15291
Colon Transverse	65 (13)	3.23E-06	15500	62 (8)	3.22E-06	15507	5 (1)	3.19E-06	15681
Esophagus Gastroesophageal Junction	71 (6)	3.34E-06	14988	66 (6)	3.33E-06	14993	5 (1)	3.30E-06	15140
Esophagus Mucosa	66 (21)	3.27E-06	15268	63 (18)	3.27E-06	15275	7 (1)	3.24E-06	15437
Esophagus Muscularis	68 (10)	3.29E-06	15199	86 (20)	3.29E-06	15205	4 (1)	3.26E-06	15343
Heart Atrial Appendage	71 (16)	3.36E-06	14879	75 (7)	3.36E-06	14890	8 (1)	3.33E-06	15030
Heart Left Ventricle	63 (14)	3.43E-06	14558	70 (15)	3.43E-06	14569	5 (2)	3.40E-06	14696

Liver	68 (11)	3.49E-06	14325	66 (12)	3.49E-06	14337	4 (2)	3.45E-06	14497
Lung	76 (20)	3.16E-06	15813	65 (10)	3.16E-06	15822	4 (1)	3.13E-06	15974
Muscle Skeletal	63 (17)	3.43E-06	14560	58 (16)	3.43E-06	14564	5 (2)	3.40E-06	14696
Nerve Tibial	69 (25)	3.22E-06	15548	69 (21)	3.21E-06	15559	5 (2)	3.18E-06	15706
Ovary	72 (2)	3.39E-06	14754	76 (10)	3.39E-06	14760	4 (1)	3.36E-06	14898
Pancreas	66 (16)	3.36E-06	14891	56 (14)	3.36E-06	14900	4 (2)	3.33E-06	15026
Pituitary	74 (7)	3.22E-06	15517	75 (10)	3.22E-06	15530	4 (1)	3.19E-06	15694
Prostate	70 (3)	3.24E-06	15420	67 (6)	3.24E-06	15429	6 (1)	3.21E-06	15588
Skin Not Sun Exposed Suprapubic	68 (13)	3.22E-06	15545	69 (13)	3.21E-06	15555	3 (2)	3.18E-06	15735
Skin Sun Exposed Lower leg	70 (19)	3.18E-06	15729	66 (25)	3.18E-06	15738	4 (2)	3.15E-06	15891
Small Intestine Terminal Ileum	70 (7)	3.28E-06	15265	61 (10)	3.27E-06	15281	2 (1)	3.23E-06	15462
Spleen	70 (13)	3.36E-06	14873	56 (9)	3.36E-06	14884	6 (1)	3.33E-06	15037
Stomach	65 (7)	3.23E-06	15495	64 (12)	3.22E-06	15506	4 (1)	3.19E-06	15658
Testis	64 (14)	3.03E-06	16520	72 (17)	3.03E-06	16528	4 (1)	2.98E-06	16764
Thyroid	63 (17)	3.18E-06	15705	57 (16)	3.18E-06	15714	7 (5)	3.15E-06	15876
Uterus	66 (7)	3.42E-06	14641	74 (3)	3.41E-06	14654	5 (1)	3.38E-06	14803
Vagina	66 (2)	3.30E-06	15157	74 (1)	3.30E-06	15167	3 (1)	3.26E-06	15328
Whole Blood	68 (26)	3.49E-06	14331	55 (11)	3.49E-06	14340	4 (1)	3.45E-06	14505
Average	66.73 (11.59)			67.70 (11.80)			4.57 (1.52)		

Supplementary Table 6 – TWAS association signals for SWAM

We used SWAM to derive an tissue-specific model for every GTEx version 6 tissue, and used these models as inputs to metaXcan to infer TWAS results. As mentioned in the methods section, the HDL and LDL traits were from Global Lipids Genetics Consortium (GLGC) and Type-2 Diabetes (T2D) from the DIAGRAM consortium. The counts in parentheses indicate the number of genes overlapping with the prediXcan results (in S7 Table).
