# Supplementary material for: Meta-imputation of transcriptome from genotypes across multiple datasets by leveraging publicly available summary-level data
Source: PLoS Genet. 2022 Jan 31;18(1):e1009571. doi: 10.1371/journal.pgen.1009571 (PMC8830793; doi:10.1371/journal.pgen.1009571)
Supplement: S7 Table — These models were also derived from GTEx version 6 tissues using the UTMOST method. Models were downloaded from https://github.com/Joker-Jerome/UTMOST (PDF) [file pgen.1009571.s016.pdf]

Tissue	HDL			LDL			T2D		
	# sig genes	p-value threshold	total genes	# sig genes	p-value threshold	total genes	# sig genes	p-value threshold	total genes
Adipose Subcutaneous	62 (16)	4.18E-06	11964	55 (5)	4.18E-06	11969	5 (2)	3.94E-06	12688
Adipose Visceral Omentum	55 (9)	4.26E-06	11741	55 (4)	4.26E-06	11743	6 (0)	4.01E-06	12475
Adrenal Gland	55 (7)	4.55E-06	10998	54 (3)	4.54E-06	11004	3 (1)	4.26E-06	11737
Artery Aorta	57 (12)	4.47E-06	11180	47 (12)	4.47E-06	11184	4 (2)	4.19E-06	11926
Artery Coronary	53 (3)	4.39E-06	11391	52 (4)	4.39E-06	11397	3 (1)	4.13E-06	12114
Artery Tibial	55 (11)	4.43E-06	11292	52 (7)	4.43E-06	11295	6 (2)	4.15E-06	12044
Brain Anterior cingulate cortex BA24	46 (3)	5.00E-06	10002	57 (2)	5.00E-06	10003	5 (0)	4.64E-06	10779
Brain Caudate basal ganglia	51 (2)	4.61E-06	10850	48 (8)	4.61E-06	10850	3 (0)	4.30E-06	11627
Brain Cerebellar Hemisphere	51 (4)	4.94E-06	10113	53 (7)	4.94E-06	10115	5 (2)	4.60E-06	10864
Brain Cerebellum	45 (5)	4.78E-06	10457	62 (11)	4.78E-06	10457	4 (2)	4.46E-06	11205
Brain Cortex	46 (5)	4.69E-06	10650	44 (10)	4.70E-06	10647	2 (0)	4.37E-06	11445
Brain Frontal Cortex BA9	50 (7)	4.67E-06	10717	52 (6)	4.66E-06	10719	6 (1)	4.34E-06	11519
Brain Hippocampus	52 (0)	4.67E-06	10701	46 (3)	4.67E-06	10701	4 (1)	4.36E-06	11469
Brain Hypothalamus	61 (1)	4.55E-06	10986	61 (4)	4.55E-06	10987	3 (0)	4.25E-06	11764
Brain Nucleus accumbens basal ganglia	58 (4)	4.64E-06	10781	49 (4)	4.64E-06	10783	5 (0)	4.33E-06	11541
Brain Putamen basal ganglia	55 (2)	4.84E-06	10323	51 (4)	4.84E-06	10327	4 (0)	4.49E-06	11129
Breast Mammary Tissue	58 (6)	4.12E-06	12141	57 (6)	4.12E-06	12143	4 (0)	3.88E-06	12899
Cells EBV-transformed lymphocytes	49 (5)	5.20E-06	9610	47 (5)	5.20E-06	9615	3 (1)	4.87E-06	10267
Cells Transformed fibroblasts	52 (17)	4.80E-06	10406	50 (11)	4.80E-06	10409	5 (2)	4.51E-06	11089
Colon Sigmoid	54 (4)	4.44E-06	11257	59 (4)	4.44E-06	11261	4 (0)	4.16E-06	12010
Colon Transverse	58 (9)	4.25E-06	11759	54 (6)	4.25E-06	11765	6 (1)	3.99E-06	12545
Esophagus Gastroesophageal Junction	48 (3)	4.50E-06	11117	42 (2)	4.50E-06	11119	2 (0)	4.23E-06	11823
Esophagus Mucosa	63 (18)	4.31E-06	11595	62 (11)	4.31E-06	11603	3 (1)	4.06E-06	12316
Esophagus Muscularis	52 (8)	4.43E-06	11285	56 (9)	4.43E-06	11289	5 (1)	4.16E-06	12029
Heart Atrial Appendage	57 (11)	4.58E-06	10922	56 (6)	4.58E-06	10923	4 (0)	4.28E-06	11679

Heart Left Ventricle	53 (12)	4.75E-06	10519	45 (6)	4.75E-06	10522	3 (0)	4.45E-06	11242
Liver	54 (7)	4.97E-06	10062	56 (6)	4.97E-06	10068	2 (0)	4.63E-06	10808
Lung	68 (12)	4.02E-06	12428	53 (3)	4.02E-06	12433	3 (2)	3.80E-06	13152
Muscle Skeletal	57 (10)	4.70E-06	10629	54 (10)	4.70E-06	10631	3 (1)	4.41E-06	11326
Nerve Tibial	58 (19)	4.24E-06	11784	57 (12)	4.24E-06	11787	4 (3)	4.00E-06	12511
Ovary	44 (0)	4.71E-06	10608	51 (5)	4.71E-06	10607	4 (2)	4.42E-06	11323
Pancreas	44 (8)	4.71E-06	10619	54 (11)	4.71E-06	10625	3 (1)	4.38E-06	11410
Pituitary	56 (4)	4.41E-06	11336	61 (5)	4.41E-06	11337	1 (0)	4.13E-06	12117
Prostate	52 (3)	4.28E-06	11685	55 (3)	4.28E-06	11688	4 (0)	4.02E-06	12450
Skin Not Sun Exposed Suprapubic	57 (7)	4.24E-06	11789	61 (8)	4.24E-06	11797	4 (2)	4.00E-06	12505
Skin Sun Exposed Lower leg	55 (16)	4.12E-06	12144	53 (15)	4.12E-06	12149	4 (2)	3.87E-06	12904
Small Intestine Terminal Ileum	50 (4)	4.48E-06	11150	59 (4)	4.48E-06	11151	3 (0)	4.19E-06	11938
Spleen	48 (9)	4.66E-06	10736	47 (5)	4.66E-06	10739	7 (1)	4.36E-06	11474
Stomach	55 (5)	4.23E-06	11833	62 (7)	4.22E-06	11838	7 (1)	3.97E-06	12580
Testis	58 (11)	4.03E-06	12411	59 (6)	4.03E-06	12412	2 (1)	3.78E-06	13222
Thyroid	62 (11)	4.13E-06	12106	56 (13)	4.13E-06	12113	7 (3)	3.88E-06	12873
Uterus	51 (4)	4.93E-06	10148	55 (0)	4.93E-06	10151	3 (1)	4.62E-06	10813
Vagina	55 (3)	4.43E-06	11285	48 (1)	4.43E-06	11288	1 (0)	4.16E-06	12018
Whole Blood	43 (12)	4.76E-06	10511	46 (10)	4.75E-06	10518	4 (1)	4.48E-06	11168
Average	53.70 (7.48)			53.48 (6.45)			3.93 (0.93)		

Supplementary Table 7 – TWAS association signals for UTMOST

These models were also derived from GTEx version 6 tissues using the UTMOST method. Models were downloaded from https://github.com/Joker-Jerome/UTMOST. The counts in parentheses indicate the number of genes overlapping with the prediXcan results (in S7 Table).
