# Supplementary material for: Meta-imputation of transcriptome from genotypes across multiple datasets by leveraging publicly available summary-level data
Source: PLoS Genet. 2022 Jan 31;18(1):e1009571. doi: 10.1371/journal.pgen.1009571 (PMC8830793; doi:10.1371/journal.pgen.1009571)
Supplement: S8 Table — TWAS results via metaXcan using prediXcan single tissue models derived from GTEx version 6 tissues (PDF) [file pgen.1009571.s017.pdf]

Tissue	HDL			LDL			T2D		
	# sig genes	p-value threshold	total genes	# sig genes	p-value threshold	total genes	# sig genes	p-value threshold	total genes
Adipose Subcutaneous	35	7.54E-06	6634	25	7.54E-06	6628	7	6.91E-06	7234
Adipose Visceral Omentum	31	1.20E-05	4174	15	1.20E-05	4173	4	1.10E-05	4542
Adrenal Gland	20	1.33E-05	3760	17	1.33E-05	3758	3	1.24E-05	4048
Artery Aorta	30	8.76E-06	5706	39	8.76E-06	5705	5	8.11E-06	6163
Artery Coronary	13	1.65E-05	3025	21	1.65E-05	3024	3	1.54E-05	3242
Artery Tibial	30	7.50E-06	6666	26	7.51E-06	6657	4	6.89E-06	7259
Brain Anterior cingulate cortex BA24	6	2.03E-05	2466	11	2.03E-05	2466	2	1.88E-05	2654
Brain Caudate basal ganglia	19	1.48E-05	3375	23	1.48E-05	3372	2	1.38E-05	3616
Brain Cerebellar Hemisphere	12	1.26E-05	3955	18	1.26E-05	3954	3	1.18E-05	4228
Brain Cerebellum	22	1.10E-05	4543	27	1.10E-05	4542	4	1.04E-05	4830
Brain Cortex	16	1.49E-05	3351	30	1.49E-05	3347	2	1.40E-05	3583
Brain Frontal Cortex BA9	12	1.66E-05	3013	14	1.66E-05	3013	1	1.56E-05	3211
Brain Hippocampus	14	2.12E-05	2362	10	2.12E-05	2362	2	1.97E-05	2534
Brain Hypothalamus	7	2.20E-05	2269	13	2.20E-05	2268	1	2.04E-05	2455
Brain Nucleus accumbens basal ganglia	11	1.70E-05	2935	16	1.70E-05	2935	1	1.60E-05	3131
Brain Putamen basal ganglia	13	1.91E-05	2620	17	1.91E-05	2620	4	1.78E-05	2807
Breast Mammary Tissue	21	1.16E-05	4292	17	1.17E-05	4288	3	1.07E-05	4655
Cells EBV-transformed lymphocytes	22	1.43E-05	3493	17	1.43E-05	3491	3	1.33E-05	3751
Cells Transformed fibroblasts	50	7.02E-06	7120	32	7.03E-06	7114	4	6.50E-06	7692
Colon Sigmoid	16	1.40E-05	3561	16	1.40E-05	3559	3	1.30E-05	3858
Colon Transverse	20	1.11E-05	4485	20	1.12E-05	4480	1	1.03E-05	4874
Esophagus Gastroesophageal Junction	13	1.45E-05	3456	10	1.45E-05	3454	3	1.34E-05	3727
Esophagus Mucosa	30	7.78E-06	6425	27	7.78E-06	6423	2	7.18E-06	6961
Esophagus Muscularis	20	8.37E-06	5971	29	8.38E-06	5966	3	7.70E-06	6493
Heart Atrial Appendage	25	1.19E-05	4187	18	1.19E-05	4185	2	1.11E-05	4501
Heart Left Ventricle	22	1.11E-05	4517	26	1.11E-05	4513	3	1.02E-05	4885
Liver	13	1.86E-05	2695	15	1.86E-05	2692	5	1.72E-05	2909

Lung	31	8.32E-06	6008	14	8.33E-06	6002	4	7.56E-06	6611
Muscle Skeletal	26	8.32E-06	6009	25	8.33E-06	6003	3	7.56E-06	6614
Nerve Tibial	38	6.60E-06	7577	35	6.61E-06	7570	3	6.13E-06	8157
Ovary	10	1.94E-05	2576	17	1.94E-05	2575	3	1.81E-05	2762
Pancreas	24	1.12E-05	4449	20	1.12E-05	4447	2	1.05E-05	4771
Pituitary	10	1.59E-05	3145	13	1.59E-05	3144	4	1.48E-05	3382
Prostate	9	2.09E-05	2389	20	2.09E-05	2389	3	1.92E-05	2609
Skin Not Sun Exposed Suprapubic	22	9.37E-06	5336	26	9.38E-06	5333	3	8.62E-06	5798
Skin Sun Exposed Lower leg	30	7.10E-06	7041	33	7.11E-06	7037	3	6.55E-06	7628
Small Intestine Terminal Ileum	16	1.97E-05	2538	15	1.97E-05	2536	3	1.83E-05	2729
Spleen	18	1.45E-05	3456	16	1.45E-05	3456	5	1.35E-05	3698
Stomach	12	1.27E-05	3945	20	1.27E-05	3943	2	1.17E-05	4271
Testis	26	7.37E-06	6780	25	7.38E-06	6778	7	6.91E-06	7234
Thyroid	28	6.69E-06	7478	34	6.69E-06	7469	6	6.14E-06	8140
Uterus	10	2.51E-05	1991	8	2.51E-05	1991	1	2.34E-05	2139
Vagina	15	2.57E-05	1946	6	2.57E-05	1944	2	2.40E-05	2082
Whole Blood	35	8.23E-06	6077	24	8.23E-06	6073	2	7.42E-06	6743
Average	20.52			20.45			3.09		

Supplementary Table 8 – *TWAS association signals for prediXcan (single-tissue)*

TWAS results via metaXcan using prediXcan single tissue models derived from GTEx version 6 tissues
