# Supplementary material for: Meta-imputation of transcriptome from genotypes across multiple datasets by leveraging publicly available summary-level data
Source: PLoS Genet. 2022 Jan 31;18(1):e1009571. doi: 10.1371/journal.pgen.1009571 (PMC8830793; doi:10.1371/journal.pgen.1009571)
Supplement: S9 Table — To control for false-positives, we calculated the genomic inflation factor for every TWAS dataset and used these to adjust the significance threshold for each analysis accordingly. (PDF) [file pgen.1009571.s018.pdf]

Tissue	HDL			LDL			T2D		
	PrediXcan	UTMOST	SWAM	PrediXcan	UTMOST	SWAM	PrediXcan	UTMOST	SWAM
Adipose Subcutaneous	1.179	1.283	1.141	1.142	1.158	1.153	1.255	1.348	1.225
Adipose Visceral Omentum	1.043	1.231	1.127	1.150	1.169	1.124	1.123	1.309	1.217
Adrenal Gland	1.132	1.287	1.155	1.116	1.186	1.142	1.068	1.322	1.209
Artery Aorta	1.132	1.254	1.143	1.195	1.179	1.121	1.100	1.350	1.192
Artery Coronary	1.037	1.204	1.147	1.027	1.188	1.152	0.999	1.333	1.188
Artery Tibial	1.132	1.299	1.163	1.137	1.164	1.129	1.197	1.324	1.210
Brain Anterior cingulate cortex BA24	1.036	1.247	1.095	1.061	1.123	1.104	1.028	1.352	1.170
Brain Caudate basal ganglia	1.052	1.224	1.139	1.119	1.145	1.092	1.062	1.313	1.180
Brain Cerebellar Hemisphere	1.043	1.250	1.107	1.047	1.192	1.107	1.064	1.353	1.175
Brain Cerebellum	1.033	1.262	1.150	1.028	1.152	1.108	1.012	1.331	1.159
Brain Cortex	1.072	1.233	1.158	1.084	1.173	1.071	1.047	1.315	1.156
Brain Frontal Cortex BA9	1.056	1.218	1.138	1.203	1.105	1.079	1.047	1.327	1.152
Brain Hippocampus	0.930	1.190	1.101	1.048	1.188	1.104	0.892	1.292	1.171
Brain Hypothalamus	0.963	1.211	1.129	0.904	1.116	1.053	1.003	1.331	1.166
Brain Nucleus accumbens basal ganglia	1.126	1.214	1.163	1.110	1.157	1.081	1.045	1.298	1.193
Brain Putamen basal ganglia	1.050	1.253	1.138	1.014	1.145	1.068	1.043	1.284	1.172
Breast Mammary Tissue	1.150	1.210	1.150	1.170	1.135	1.124	1.141	1.316	1.250
Cells EBV-transformed lymphocytes	1.008	1.240	1.148	1.032	1.207	1.174	1.047	1.327	1.266
Cells Transformed fibroblasts	1.115	1.263	1.175	1.116	1.166	1.154	1.234	1.362	1.235
Colon Sigmoid	0.988	1.290	1.161	1.062	1.196	1.121	1.094	1.305	1.179
Colon Transverse	1.185	1.244	1.133	1.159	1.186	1.150	1.191	1.308	1.186
Esophagus Gastroesophageal Junction	1.037	1.214	1.131	1.152	1.187	1.144	1.060	1.348	1.183
Esophagus Mucosa	1.148	1.192	1.160	1.127	1.204	1.149	1.126	1.351	1.229
Esophagus Muscularis	1.184	1.224	1.171	1.161	1.214	1.117	1.138	1.295	1.169
Heart Atrial Appendage	1.166	1.244	1.145	1.143	1.175	1.148	1.092	1.369	1.173
Heart Left Ventricle	1.173	1.266	1.146	1.161	1.219	1.136	1.164	1.335	1.195

Liver	1.088	1.234	1.160	1.146	1.189	1.149	0.987	1.308	1.191
Lung	1.125	1.218	1.141	1.134	1.169	1.135	1.111	1.292	1.171
Muscle Skeletal	1.176	1.263	1.184	1.136	1.176	1.151	1.230	1.373	1.214
Nerve Tibial	1.105	1.277	1.159	1.131	1.180	1.155	1.181	1.306	1.220
Ovary	1.089	1.242	1.145	1.004	1.181	1.099	1.040	1.295	1.212
Pancreas	1.121	1.279	1.192	1.172	1.170	1.154	1.162	1.340	1.228
Pituitary	1.054	1.259	1.133	1.018	1.159	1.098	1.029	1.340	1.175
Prostate	1.047	1.219	1.157	1.017	1.175	1.145	0.968	1.298	1.226
Skin Not Sun Exposed Suprapubic	1.158	1.216	1.151	1.106	1.150	1.138	1.114	1.334	1.192
Skin Sun Exposed Lower leg	1.188	1.266	1.176	1.118	1.225	1.153	1.241	1.339	1.229
Small Intestine Terminal Ileum	0.940	1.237	1.132	1.046	1.165	1.110	0.967	1.329	1.183
Spleen	1.034	1.237	1.149	1.065	1.130	1.152	1.018	1.297	1.244
Stomach	1.134	1.231	1.149	1.105	1.141	1.141	1.124	1.312	1.227
Testis	1.093	1.222	1.175	1.113	1.177	1.123	1.014	1.309	1.212
Thyroid	1.164	1.251	1.194	1.154	1.149	1.170	1.203	1.319	1.176
Uterus	1.106	1.179	1.192	1.046	1.138	1.118	0.989	1.311	1.194
Vagina	0.979	1.245	1.150	1.108	1.167	1.110	0.939	1.292	1.213
Whole Blood	1.164	1.284	1.130	1.169	1.203	1.163	1.214	1.348	1.201

Supplementary Table 9 – Genomic inflation factor for every TWAS dataset

To control for false-positives, we calculated the genomic inflation factor for every TWAS dataset and used these to adjust the significance threshold for each analysis accordingly.
