# Supplementary material for: Meta-imputation of transcriptome from genotypes across multiple datasets by leveraging publicly available summary-level data
Source: PLoS Genet. 2022 Jan 31;18(1):e1009571. doi: 10.1371/journal.pgen.1009571 (PMC8830793; doi:10.1371/journal.pgen.1009571)
Supplement: Attachment — Submitted filename: SWAM response to reviewers.docx [file pgen.1009571.s019.docx]

**Response to Reviewer Comments**

**Manuscript ID : PGENETICS-D-21-00570**

**Title : Meta-imputation of transcriptome from genotypes across multiple datasets using summary-level data**

**We thank the reviewers and editors for their thoughtful and constructive comments that have substantially improved the manuscript. Below are the reviewer comments (in italics) and out detailed responses to each (in bold)**

**Reviewer #1 Comments to the Author:**

*The authors presents a very interesting and useful approach to aggregate reference data of multiple tissues and cohorts for TWAS. I have the following major comments:*

*1. The authors use the same data set to learn model weights and test accuracy. The test results will be “over fitted” because the Optimal Model Weights were learned using the same data set. The authors would need to either use another independent data set as test data or split the current test data into validation data for training model weights and an independent test data. This should also be done for all simulation studies.*

**Thank you for raising an important point on SWAM and we apologize for lack of clarity in the original version manuscript. In fact, our evaluation did not use the same dataset for evaluating test accuracy. We never use GEUVADIS LCL data for learning model weights so that GEUVADIS can be used as a test dataset without overfitting. In our empirical validation, we trained our SWAM models using GTEx tissues. We used individual-level data only for target tissue, which is EBV-transformed lymphocytes in GTEx, not GEUVADIS LCL. Therefore, there will be no systematic overfitting in our results. We used individual-level RNA-seq measured expression from the GEUVADIS consortium only for testing purpose.**

**In our simulation studies, we generated two independent sets of data: one to train SWAM and one to test its imputation accuracy, and the test datasets were never used for learning weights for SWAM.**

**We understand that readers may have been confused with our original description, and we revised our manuscript to clarify the terms. Specifically, we modified the following paragraphs in our revised manuscript to improve the clarity on how training and evaluation was performed:**

**[Results]** As such, our method only requires individual-level genotypes and expression for the reference tissue, and then integrates imputation models that were already trained from different tissues and datasets (e.g. GTEx, DGN, and TCGA). (Lines 153-156)

**[Results]** We performed simulation studies to evaluate SWAM’s ability to robustly impute expression by leveraging tissue-specific and cross-tissue components across a wide spectrum of parameter settings. To do this, we independently simulated multi-tissue expression levels along with genotype data for both our training and validation sets (see Materials and Methods). We used the training set solely to derive SWAM models, while the validation sets were used to test our models by comparing the imputed expression with the actual (simulated) expression. We compared SWAM with two heuristic approaches – *naïve average,* which equally weights individual tissue and *best tissue,* which only uses the tissue with the highest expected imputation accuracy – as well as with *single-tissue* imputation. (Line 168-176)

**[Results]** We used SWAM to integrate all 49 PrediXcan models trained from each tissue using GTEx v8 release to generate a multi-tissue model. Except for the target tissue LCL (lymphoblastoid cell line tissue or “Cells – EBV-transformed lymphocytes”), individual-level genotypes and expression levels were not used to build the SWAM model. We then externally validated the imputation accuracy of this GTEx-based SWAM model by applying it to impute expression for 344 European LCL samples from the GEUVADIS consortium [29], and compared this to the measured expression levels of the corresponding individuals. (Lines 222-228)

**[Results]** We also evaluated the performance of alternative methods including (1) single-tissue, (2) naïve average, (3) best-tissue, and (4) the *UTMOST* method. For single tissue, we repeated this validation for each of the 49 GTEx v8 single tissue­ imputation models generated by PrediXcan. Naive average and best tissue, our other heuristic approaches, were also evaluated by using the GTEx LCL as the target tissue. We also evaluated another multi-tissue method *UTMOST* [24]*.* Because *UTMOST* models were built based on GTEx v6, we used v6 for any evaluations involving *UTMOST* (Lines 229-235)

**[Results]** The key component behind the robust performance of SWAM is that it learns how to distribute weights across multiple imputation models for each gene individually. If a gene shares eQTLs across many tissues, the SWAM’s weights will be distributed evenly across tissues and the model will behave similarly to the naïve average heuristic. For example, *ERAP2* is a well-known gene with shared eQTLs profiles across most tissues. In GTEx v8, *ERAP2* can be reliably imputed with any of the 49 single-tissue imputation models from PrediXcan with r^2^ > 0.77 or higher. As a result, the weights from SWAM are almost evenly distributed across the tissues, ranging from 0.006 (0.01 excluding LCL) to 0.031 (S4 Fig), and the accuracy of SWAM (r^2^ = 0.812) is very similar to the accuracy of naïve average (r^2^ = 0.811). (Lines 308-316)

On the other hand, when the imputation model from the reference tissue is not particularly good due to smaller sample size or other technical issues, SWAM can substantially improve accuracy by leveraging eQTL sharing from other tissues. For example, the single-tissue imputation accuracy of *GSTM4* is relatively low in LCL tissue (r^2^ = 0.125) compared to the accuracy of the 38 other tissues in which a PrediXcan imputation model is available (average r^2^ = 0.307). Using SWAM, leverages 39 tissues by assigning low weight to the LCL tissue, and higher weights to more relevant tissues, which thereby increases the predictive R-squared to r^2^ = 0.492 (S4 Fig). (Lines 318-324)

Finally, for genes that are highly tissue-specific, the SWAM’s weights will be distributed similarly to the best tissue heuristic. For example, *PTBP3* is expressed in most tissues, but has significant eQTL signals in only 15 tissues. SWAM assigns weights to 9 of these tissues, and substantially improves the predictive accuracy from r^2^ = 0.111 to r^2^ = 0.447 (S4 Fig). (Lines 325-328)

**[Supplementary Figure 4]** We replaced this figure with an updated one with GTEx v8 tissues

*2. The simulation study should be better designed to mimic real TWAS. Modeling only 1 or 2 true causal eQTL is not realistic for TWAS as the authors can see how many number of SNPs would be tested by PrediXcan using GTEx reference data. Also, the authors only consider independent SNPs in the simulation studies, which is not realistic as the gene expression imputation models are trained using all cis-SNPs that are in LD and are of about thousands per gene.*

**Thank you for pointing that our simulation analysis does not reflect realistic settings of TWAS where many causal variants might be involved. We completely agree with the reviewer that our simulation settings do not fully reflect the real-world examples on how eQTLs affect complex traits and GWAS results. In fact, we believe that it is extremely challenging to comprehensively account for possible real-world scenario of molecular QTLs affecting complex traits, so our manuscript focused on illustrating the practical utility of our method using the real data from GTEx, DGN, and GEUVADIS data quite extensively. The main purpose of our simulation study is to illustrate that SWAM behaves as expected in a rather simplistic scenario where we know what is expected to happen.**

**However, we believe that it is still important to expand our simulation study to account for more complicated scenario, even though it will be challenging to be fully comprehensive. To this end, we expanded our simulation results to encompass the examples with more causal SNPs and replaced our results in Figure 2 based on 10 causal SNPs, and moved the original results based on 2 causal SNPs in Figure S7. As described in the revised manuscript, these results are very similar to each other. We believe that this is because the expected imputation accuracy largely depends on the total heritability explained by the causal SNPs. To make this point, we further evaluated the performance of SWAM across various number of causal variants (5 to 125), and showed that the expected imputation accuracies remain very similar across these parameters (Figure S2). We revised our manuscript on the simulation study to reflect this change as follows:**

**[Results section]** Finally, we investigated the performance of our approaches over different levels of heritability (Figure 2E), and across a wide spectrum of p-values (Figure 2F), confirming that SWAM outperforms the other methods across these parameter settings. To better reflect more complex and realistic scenarios typically found in eQTL data, we also expanded our simulations to evaluate the impact of a higher number of causal SNPs on predictive performance of SWAM (S3 Fig). Here, we varied the number of causal SNPs from 5 to 125 and examined the performance of SWAM across a wide range of heritability levels. We found that the number of causal variants did not impact the performance of SWAM, with predictive R^2^ remaining the same when increasing the number of variants. (Line 211-219)

**[Methods section]:** For reference tissue (i.e.$j=r$), we assume ten causal SNPs with nonzero elements in $\beta_{j}$, where five SNPs is expected to explain tissue-specific heritability ($h_{r}^{2}$) for the reference tissue and the other five SNPs explain the cross-tissue heritability ($h_{c}^{2}$), summing up to total heritability ($h^{2}=h_{r}^{2}+h_{c}^{2}$) (Line 502-505)

**[Methods section]:** We then expanded our simulation to include a larger number of causal variants, ranging from 5 to 125 SNPs across a spectrum of heritability levels. For these simulations, we kept most the same parameter settings as our other simulations except for generating 5,000 individuals as opposed to the default 200. **(Line 524-527)**

**[Supplementary materials]:** we added Figure S2 to show the impact of an increased number of causal variants for our simulation studies (page: 52)

*3. I do not think the authors need to discuss using all versions of GTEx data as the most recent GTEX V8 would contain all samples from the previous versions. I think the authors should focuse on using GTEx V8 data in this paper.*

**Thank you for this suggestion, and we totally agree that it would be ideal to apply the method to the latest GTEx V8 data. However, since the published UTMOST model was produced based on GTEx v6, and it is always tricky to apply someone else’s method on the new data for the evaluation of our own method, we believed that it is most straightforward if we took the model trained by authors of UTMOST method when we evaluate SWAM and UTMOST. For a fair comparison, we had to use GTEx v6 in the portion of the analysis that required comparison with UTMOST.**

**However, as the reviewer suggested, there is no reason to use GTEx v6 when we evaluate SWAM in other contexts. Therefore, we expanded our figures, tables, and corresponding texts to present results from GTEx v8 too and refer to GTEx v8 whenever comparison with UTMOST was not the primary point. To reflect these changes, we revised our current manuscript in multiple places, including the following changes:**

- **We have defaulted to GTEx version 8 whenever possible, and have included an explanation as to why we use GTEx version 6 for some of our analyses involving UTMOST. (See above. Line 222-228)**
- **We modified our single-tissue evaluation to reflect results GTEx v8 tissues. For example, the number of significantly imputable genes at FDR < 0.05 increased from 1,552 (v6) to 1,620 (v8) for LCL (See line 236-258 for the full paragraph).**
- **We explicitly mention GTEx v6 when comparing multi-tissue results involving UTMOST in our results section: (lines 259-271)**

**[Results]** When evaluating the multi-tissue methods, our two heuristic approaches (both using GTEx v6 tissues), best-tissue and naïve average identified 2,493 and 2,666 significantly imputable genes, respectively, which was >47% and >57% higher than any of the 44 GTEx v6 single tissue models. UTMOST (using LCL as the reference) also substantially increased the number of imputable genes (2,238 genes, >32% increase over any single tissue from GTEx v6), but surprisingly, it had fewer than the imputable genes compared to the two heuristic approaches. Finally, when we applied SWAM specifying GTEx-v6 LCL as the reference tissue…

*4. The authors should discuss the proportion of overlapped significant TWAS genes by Single Tissue PrediXcan, UTMOST and SWAM method, and the number of genes that only identified by UTMOST or SWAM method, or single tissue PrediXcan.*

**Thanks for the excellent suggestion. We agree that this comparison will highlight the differences between the methods clearly, and we included counts of the overlapping signals between SWAM and PrediXcan in Supplementary Table 5, and UTMOST and PrediXcan in Supplementary Table 6. We also updated the Manhattan plot in Figure 4 to visually highlight which genes are found SWAM but not in other methods, including *APOC1*, *NYNRIN*, and *SORT1*. We have also updated the manuscript (lines 344-356) to discuss these overlaps.**

**[Results]** We also examined the number of replicated signals between the two multi-tissue approaches (SWAM, UTMOST) and the single-tissue method (PrediXcan). We found that on average, 17.4%, 17.4% and 33.3% of SWAM’s signals were replicated in their single-tissue counterpart for HDL, LDL and T2D respectively. For UTMOST, these replication rates were 13.9%, 12.1% and 23.7% for HDL, LDL and T2D. The higher overlap in signals for SWAM models may be a result of higher tissue-specificity compared to the UTMOST models.

We plotted transcriptome-wide signals for the LDL trait using the GTEx v6 liver model for PrediXcan, UTMOST and SWAM (Figure 4). One interesting signal gained from the SWAM analysis is the *APOC1* gene, which is primarily expressed in the liver and has been implicated in playing a role in HDL and LDL/VLDL (very low-density lipid) metabolism [31]. For this trait-tissue combination, every gene replicated between UTMOST and PrediXcan was also replicated between SWAM and PrediXcan. In addition, the *SORT1* gene, which has been found to be associated with LDL-C levels, was only detected in SWAM and PrediXcan [32]. (Lines 344-356)

*Reviewer #2: Liu and Kang proposed SWAM (Smartly Weighted Averaging across Multiple datasets) to improve the gene expression imputation of the target tissue using pre-trained imputation models from other relevant tissues. The weight for each tissue depends on the correlation among tissues, the correlation between imputed and observed expression in the testing set, and a penalty tuning parameter. They conducted simulations and real data analyses to demonstrate the advantage over existing methods in imputation accuracy and the number of genes identified in transcriptome-wide association studies (TWAS). Below are some comments that may improve the manuscript.

The title “Meta-imputation of transcriptome from genotypes across multiple datasets using summary-level data” gave the impression that the proposed method only requires summary-level data. However, this is not true according to the following excerpt.
“Using the individual level genotypes and expression of only the reference tissue, SWAM integrates imputation models trained from different tissues and datasets without requiring individual-level data except for the reference tissue.”
So “using summary-level data” is a little bit misleading. The proposed method SWAM does require the individual-level expression of the reference tissue to calculate weights to impute gene expression in the target tissue. Only if the authors can upload trained weights in a way similar to predictDB or FUSION, the method would be fully based on summary-level data. Otherwise, the authors may need to clarify this potential misunderstanding.*

**Thank you for pointing out the lack of clarity in our current title. We completely agree that the title could be misinterpreted, and in the revised manuscript, we modified the title as “Meta-imputation of transcriptome from genotypes across multiple datasets by leveraging publicly available summary-level data”. To avoid misinterpretation, we also made it clear in our abstract to mention that our method requires individual-level data for the tissue of interest to maximize the imputation accuracy as quote in the following text: (Lines 34-36)**

**[Abstract]** By integrating models across tissues and/or individuals, SWAM can improve the accuracy of transcriptome imputation or to improve power to TWAS while only requiring individual-level data from a single reference cohort. (Lines 34-36)

*SAWM relies on a tuning parameter lambda to obtain stable meta weights. Right now, it was empirically set as 3. Can the tuning parameter lambda be selected via cross-validation?*

**Thank you for raising an important point. The primary reason we set the regularization parameter lambda to a constant value was because our evaluation in the original manuscript (Supplementary Figure 5) showed that the accuracy remains very similar across a wide range of the parameter. However, we agree that it is important that the users should be able to tweak the parameters or re-trained the parameter as they need. Therefore, we modified our software to allow users to select the regularization parameter through cross-validation. We also modified our manuscript to reflect this point clearly in our Discussion and Methods section as follows. (select tuning parameter across all genes, not gene by gene) – think it would be good to show the plot of our empirical validation**

**We implemented an option in our software that allows the user to ascertain the tuning parameter through cross-validation. We have revised our manuscript in the supplementary section (lines 878-883) accordingly**

**[Supplementary Materials]** We found empirically that λ=3 provided the best results in our real-data validation of SWAM, which we use as the default value in our software (Fig S5). However, because this value may depend highly on the scale and normalization of the data, we also implemented an optional 10-fold cross-validation step in our software to empirically determine the optimal λ based on the input data. We also still provide users with an option that allows the user to manually specify the value of λ. (Lines 878-883)

**We also included a supplementary figure to demonstrate the rationale behind our default choice of tuning parameter (supplementary Figure 5) and updated the discussion section to elaborate on the choice of the parameter.**

**[Discussion]** Similarly, the choice of regularization parameter λ may need a fine-tuning depending on the correlation structure between tissues. In such cases, the SWAM software tool offers automatic selection of the parameter λ using cross-validation. Further in-depth analysis of the behavior of SWAM imputation model, which is essentially an ensemble learning method across multiple imputation models, could help determine these thresholds more efficiently without having to rely on computationally intensive procedure to select these parameters empirically. (Lines 421-427)

*It is good that SWAM identified more genes in TWAS. But it may be necessary to demonstrate that SWAM controls for false positives, either through simulations or the histogram/QQ plot of the p-values.*

**Thank you for the excellent point about potential false positives in SWAM. We agree that it is important to show that SWAM is robust against false positive while maintaining higher power. To illustrate this point, we made two changes from the original manuscript. First, we reported genomic control inflation factor (λ_GC_) for each TWAS dataset, and found that the inflation factor were very similar between single tissue TWAS (λ_GC_=1.11), UTMOST TWAS (λ_GC_=1.24), and SWAM TWAS (λ_GC_=1.15), for example in LDL. We believe that the amount of inflation is fairly modest across the tests we tested. Second, we updated our Manhattan plots and the number of significant signals in Supplementary Table S5-S7 after correcting for inflation factor using genomic control. We also produced QQ plots before/after correcting for genomic inflation and included in Supplementary Figure S8 in our revised manuscript. We also modified the following sentences in the revised manuscript to reflect these changes:**

**[Results]** To control for genomic inflation, we calculated and reported the genomic control inflation factor ($\lambda_{GC}$) for each TWAS dataset (S8 Table). We found that the inflation factor was very similar across methods, with medians of $\lambda_{GC}= 1.11$ for prediXcan, $\lambda_{GC}= 1.24$ for UTMOST and $\lambda_{GC}= 1.15$ for SWAM. (Lines 332-335)

UTMOST was compared in the real data application. Why was it not compared in the simulations?

**Thank you for raising the important point regarding the simulations. We initially considered incorporating UTMOST in our simulation study, but we had two major concerns. First is that the UTMOST requires several custom settings to calibrate the training models, and we did not feel comfortable that we are aware of the best practice to run UTMOST in an ideal setting, especially when it comes to simulated datasets. Second, while the mathematical model of UTMOST is pretty straightforward to understand, running UTMOST in actual data requires a specific series of computational set up and time-consuming procedures to fit the model for each instance of simulated data, so it was not practically feasible for us to run UTMOST in our simulation setting. This is why we also tried to avoid running UTMOST ourselves and rather wanted to compare the model built from the authors directly for a fairer comparison.**

*The numbering of figures in the main text and supplementary and tables is incorrect and confusing.
Fig 6D (should be Fig 2D) was cited for sample size, but it is actually about heritability, while 2F is about sample size. Both Figs 2E and 2F were not cited. The caption of subfigures of Fig 2 ABC was mismatched with the figures.
Line 292, Fig S3 should be cited other than S2.*

**This figure mis-numbering has been fixed, thank you for pointing these out.**

*“The software package for SWAM is available at* [*https://github.com/aeyliu/swam*](https://github.com/aeyliu/swam)*, including the datasets used in this manuscript.”
The link does not work.*

**Thank you for pointing out the important issue on the software availability. The software has been made available at this link now.**

*Some typos:
Line 160: to maximize expected mean squared error
Line 481, “each simulation was repeated for 1,000 times”, while in the caption of Fig 2, it said, “we run each simulation 10,000 times”. Which one is correct?* **(10,000 is correct and these numbers have been fixed)** *Line 793, the second equation contradicts with that in line 797

Line 115, “MultiXcan, *does not impute transcriptomes*, but performs a multi-tissue TWAS across all tissues by including each tissue-specific *imputed expression* as a predictor variable”
The bold parts contradict each other. It requires a clearer description.

The references wrongly put consortiums as the first author when it is actually listed in the middle. This problem applies to multiple cited references.

Some grammar errors:
Line 113, transcriptome imputation model A simultaneously across all tissues
Line 193, the power of SWAM and naïve average keepS increasing
Line 923, (B) depicts are scenario
Line 944, Each cell in this heatmap depict*

**Thank you very much for your detailed comments to improve the manuscript. All the typos and wording issues indicated above have been fixed in the revised manuscript.**
