# Supplementary material for: Meta-imputation of transcriptome from genotypes across multiple datasets by leveraging publicly available summary-level data
Source: PLoS Genet. 2022 Jan 31;18(1):e1009571. doi: 10.1371/journal.pgen.1009571 (PMC8830793; doi:10.1371/journal.pgen.1009571)
Supplement: Attachment — Submitted filename: SWAM response to reviewers.docx [file pgen.1009571.s020.docx]

**R**esponse to Reviewer Comments

Manuscript ID : PGENETICS-D-21-00570R1

Title : Meta-imputation of transcriptome from genotypes across multiple datasets using summary-level data

We thank the reviewers and editors for their time and effort to thoroughly review our revised manuscript and to provide us with constructive comments that have further improved the manuscript. Below are the reviewer comments (*in italics*) and out detailed responses to each (**in bold**)

Reviewer's Responses to Questions

Comments to the Authors:

*Reviewer #1: The authors have addressed most of my comments. I still think the authors shall compare with UTMOST in their simulations and real data with GTEx V8 data. As suggested by the other reviewer, the authors shall evaluate Type I Error in simulation studies instead of just showing the genetic control factor lambda values, and compare with UTMOST in simulation studies.*

**Thank you for your comments on the Type I error rate and additional evaluations against UTMOST. We agree that they are all important, and we performed several additional experiments to address these points, as described below.**

**First, we performed additional simulations to show that SWAM has robust Type I error rate. We simulated cases where both training and test data follows the null distribution, as well as the cases where only test data follows the null distribution. In both experiments, the Type I error rate for SWAM is well controlled, and the results are included in Supplementary Table 1 and described in line 187-188 as follows in our revised manuscript:**

When we evaluate these methods under the null model, the Type I error rates were well controlled across all five methods (S1 Table).

**Regarding Type I error rates, we would also like to make an additional note on the genomic control factor lambda. We believe that high lambda is due to the pervasive LD between causal variants and many imputed gene expression levels with large sample sizes. We included these aspects as an additional paragraph in the Discussion section for clarification as follows (line 450-458):**

While SWAM did not demonstrate inflation of false positives in our simulation, the 450 genomic control inflation factor lambda for LDL, HDL, and T2D were higher than 1.0 across all 451 TWAS methods we evaluated. We believe that this is not because of false positives but due to 452 combined effect of large sample size and linkage disequilibrium (LD) between causal variants 453 and imputed expression levels. In principle, the LDSC method [36] can help disentangle the 454 effect from false positives and pervasive LD if it supported results from TWAS association using 455 the intercept term. We note that this topic of quantifying the true inflation of TWAS statistics 456 by accounting for LD score as a future research topic of interest.

**Second, we added comparison with UTMOST in our evaluation of simulated data as suggested by the reviewer. We would like to make a note that UTMOST software tool is not written in a way that is easy to reproduce, and many people were struggling to reproduce their results (see** [**https://github.com/Joker-Jerome/UTMOST/issues/13**](https://github.com/Joker-Jerome/UTMOST/issues/13)**) and some suggestions are made by the authors (see** [**https://github.com/Joker-Jerome/UTMOST/issues/12**](https://github.com/Joker-Jerome/UTMOST/issues/12)**). We investigated these suggestions in addition to the newer segment of code available in another repository** [**https://github.com/Joker-Jerome/utmost_update**](https://github.com/Joker-Jerome/utmost_update)**. We believe that we followed the best practice authors recommend. Our code to perform the simulation experiment is transparently shared at** [**https://github.com/aeyliu/SWAM-manuscript**](https://github.com/aeyliu/SWAM-manuscript) **so that anyone can provide feedback if there is a room for improvement. Based on the results, we believe that the performance of UTMOST is consistent to the performance we observed in the real datasets and SWAM continues to outperform UTMOST in most simulation settings.**

**Third, we also performed evaluation of UTMOST results based on GTEx v8 data as suggested. Previously we avoided performing such an analysis because running someone else’s method ourselves may have a risk of not exactly following the best practice that the authors of the method would have recommended. Fortunately, we realized that GTEx v8 model trained by UTMOST authors were available at** [**http://zhaocenter.org/UTMOST**](http://zhaocenter.org/UTMOST)**, and we downloaded them and performed the evaluation. However, to our surprise, we observed that the UTMOST-GTEx v8 models they provided are far worse than any other models, including single-tissue models, UTMOST models based on GTEx v6, and SWAM models. There were only ~30 significantly imputable genes while others typically can impute thousands. We carefully examined these results and concluded that the currently available UTMOST GTEx v8 models were flawed as their prediction models had extremely poor overlaps with known eQTLs, unlike other prediction models. For example, in the GTEx v6-based UTMOST imputation model for *ERAP2* (one of the strongest imputable genes) 135 SNPs were included in the model and 41 are also included in SWAM models. However, in the GTEx v8-based UTMOST imputation model, only 4 SNPs were included in the *ERAP2* model and none of the SNPs were shared with GTEx v6-based UTMOST models. We tried to follow-up this issue with authors at** [**https://github.com/Joker-Jerome/UTMOST/issues/24**](https://github.com/Joker-Jerome/UTMOST/issues/24)**, but received no responses to date. As we believe that this problem is a result from a bug that could be fixed sometime soon, we concluded that it is the best to leave our current evaluation of GTEx results as is (i.e. based on GTEx v6). We decided not to mention this issue in our revised manuscript as we believe that this issue may be temporary. However, we provide these details in our response to reviewers to demonstrate that we tried our best to reflect the suggestion.**

Reviewer #2: My comments have been addressed.

**Thank you for your time and effort in reviewing our revised manuscript.**
